# Association between osteoporosis and rotator cuff tears: evidence from causal inference and colocalization analyses

**DOI:** 10.1038/s41413-025-00450-z

**Published:** 2025-08-28

**Authors:** Yibin Liu, Rong Zhao, Zhiyu Huang, Feifei Li, Xing Li, Kaixin Zhou, Kathleen A. Derwin, Xiaofei Zheng, Hongmin Cai, Jinjin Ma

**Affiliations:** 1 School of Medicine, South China University of Technology, Guangzhou, Guangdong China; 2 Department of Sports Medicine, The First Affiliated Hospital, Guangdong Provincial Key Laboratory of Speed Capability, The Guangzhou Key Laboratory of Precision Orthopedics and Regenerative Medicine, Jinan University, Guangzhou, Guangdong China; 3 Division of Cell, Developmental and Integrative Biology, School of Medicine, South China University of Technology, Guangzhou, Guangdong China; 4 School of Public Health, Guangzhou Medical University, Guangzhou, China; 5 Department of Biomedical Engineering, Cleveland Clinic, Cleveland, OH USA; 6 School of Computer Science and Engineering, South China University of Technology, Guangzhou, Guangdong China; 7 Institute of Future Health, South China University of Technology, Guangzhou, Guangdong China

**Keywords:** Calcium and phosphate metabolic disorders, Pathogenesis, Bone

## Abstract

Osteoporosis is a known risk factor for rotator cuff tears (RCTs), but the causal correlation and underlying mechanisms remain unclear. This study aims to evaluate the impact of osteoporosis on RCT risk and investigate their genetic associations. Using data from the UK Biobank (*n* = 457 871), cross-sectional analyses demonstrated that osteoporosis was significantly associated with an increased risk of RCTs (adjusted OR [95% CI] = 1.38 [1.25–1.52]). A longitudinal analysis of a subset of patients (*n* = 268 117) over 11 years revealed that osteoporosis increased the risk of RCTs (adjusted HR [95% CI] = 1.56 [1.29–1.87]), which is notably varied between sexes in sex-stratified analysis. Causal inference methods, including propensity score matching, inverse probability weighting, causal random forest and survival random forest models further confirmed the causal effect, both from cross-sectional and longitudinal perspectives. A colocalization analysis across multiple datasets identified six candidate loci, including the successfully replicated *PKDCC* rs12996954 variant, which may help explain the shared genetic basis between osteoporosis and RCTs. In conclusion, osteoporosis significantly increases the risk of RCTs, emphasizing the importance of osteoporosis management in preventing RCTs. The identification of shared genetic loci provides new insights into their potential pathogenic mechanisms.

## Introduction

Rotator cuff tears (RCTs) are the leading cause of shoulder pain and disability, affecting ~20%–30% of individuals aged 60 years and older, imposing a considerable societal burden.^1^ It is estimated that approximately half of patients with RCTs progress over time, and about 5% of these become symptomatic enough to require rotator cuff repair (RCR).^2^ Furthermore, 25.3% of the patients on average who undergo RCR experience retears in 2 years,^3^ causing continued pain, reduced strength, and increased shoulder disability.^4,5^ The high prevalence and progressive pathology of RCTs highlight the urgent need for a comprehensive understanding of risk factors and underlying mechanisms to develop optimized prevention and treatment strategies. However, beyond well-established risk factors such as family history, age, hand dominance, and overuse, few studies have explored causal roles of risk factors for RCTs. Overall, knowledge of risk factors contributing to the etiology and pathology of RCTs remains limited.

Osteoporosis, a prevalent bone health condition affecting 200 million people globally,^6^ is characterized by gradual bone loss, leading to increased fragility and fractures risk. The clinical association between osteoporosis and RCTs has drawn significant research interest. To date, only one large cohort study based on local medical insurance system, involving 21 066 participants, has reported an association between osteoporosis and an increased risk of RCTs over a 7-year follow-up period.^7^ This study’s substantial sample size demonstrated a strong association between osteoporosis and RCT risk. However, the analysis was limited to a single population, and further investigation is needed to better understand the relationship between these conditions. Most of other observational studies with smaller sample sizes have focused primarily on the potential effect of osteoporosis on healing outcomes in patients undergoing RCR.^8–10^ The role of osteoporosis as a risk factor for RCT incidence requires further validation in large-scale prospective cohort across diverse ethnic backgrounds.

Observational studies can only establish associations and suggest potential causal effects between traits.^11^ However, ethical and practical limitations make randomized controlled trials unfeasible for confirming the causal effect of a risk factor on RCTs. This limitation poses a significant challenge in translating clinical associations into causal relationships in current research on RCT etiology, highlighting the need for alternative approaches. In recent decades, causality inference methods have been developed to approximate true causal relationships between traits by controlling for confounding factors in epidemiological research. For example, propensity score matching (PSM) and inverse probability weighting (IPW) use parametric techniques to balance covariates between treatment (osteoporosis exposure) and control groups, reducing confounding and improving estimation of causal effects in observational studies. Additionally, machine learning (ML) algorithms such as causal random forest (CRF) and survival random forest (SRF), which are derived from generalized random forests, estimate heterogeneous treatment effects across individuals or subgroups, facilitating causality inference through causal regression methods.^12,13^

Bioinformatic approaches have also advanced, particularly with the expansion of genome-wide association studies (GWAS) to interpret the clinical associations and identify potential mechanisms between traits. Linkage disequilibrium score regression (LDSC) analysis is a linear method utilizing GWAS summary statistics to estimate the overall genetic correlations between traits by accounting for single nucleotide polymorphism (SNP)-trait associations and SNP–SNP linkage disequilibrium,^14^ suggesting the degree of sharing of genetic mechanisms. Colocalization analysis integrates GWAS summary statistics to identify shared causal variants between traits through Bayesian testing, with colocalized loci indicating shared genetic regulatory mechanisms, establishing genetic relationships that explains potential clinical associations.^15,16^

**Fig. 1:**
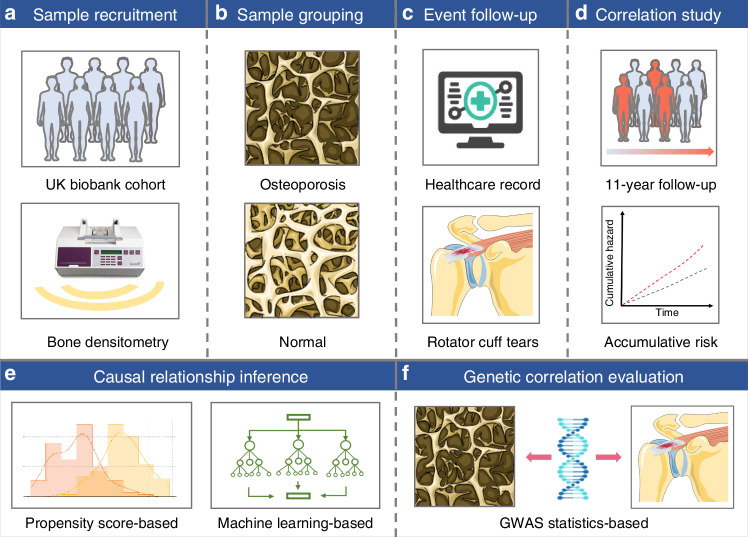
Graphical abstract of the study design. **a** Correlation analyses between osteoporosis and rotator cuff tears (RCTs) were conducted using data from the UK Biobank. **b** Samples were grouped into the osteoporosis and control groups based on bone densitometry measurements and medical records. **c** RCT events were tracked through the healthcare system. **d** Correlation analyses were performed to assess the impact of osteoporosis on RCT risk over an 11-year follow-up period. **e** Propensity score-based and machine learning-based methods were applied to infer the causal relationship between osteoporosis and RCTs. **f** Genome wide association study (GWAS) statistics-based approaches were used to evaluate the genetic correlation between osteoporosis and RCTs

Given limited research on the association between osteoporosis and RCTs, conducting large-scale prospective cohort studies across diverse populations is essential for robust conclusions. Leveraging causality inference and bioinformatics approaches can deepen our understanding of clinical correlations and provide insights into underlying mechanisms. Therefore, as shown in Fig. 1, we conducted this comprehensive study using: (1) prospective cohort analyses to estimate the association between osteoporosis and RCT risk in the UK Biobank, (2) causality inference to establish causality of osteoporosis on RCTs based on multifarious methods, including the PSM, IPW, CRF, and SRF; and (3) bioinformatics analyses to estimate genetic correlations between osteoporosis and RCTs employing LDSC analysis and colocalization analysis with publicly available GWAS summary statistics.

## Results

### Demographic characteristics of subjects from the UK Biobank cohort

**Fig. 2:**
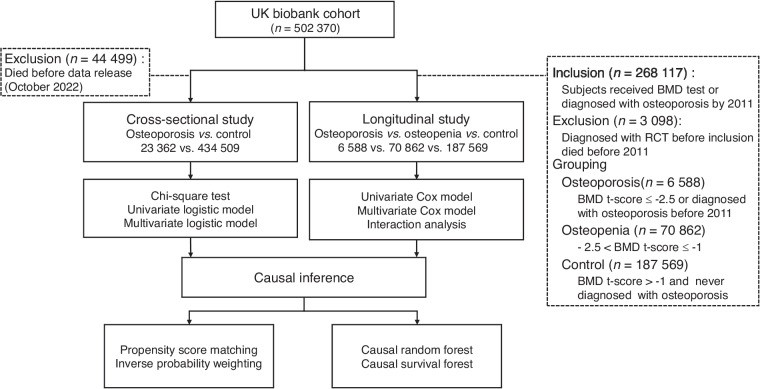
Flowchart of subjects’ screening and the analytical process

Figure 2 presents a flowchart detailing the subject screening and analytical process followed. A total of 457 871 subjects were included in the cross-sectional analysis, comprising 202 862 males and 255 009 females. Of the participants, 93.9% were of White ethnicity, with an average age of 69.79 ± 8.07 years (range: 50–88) as of October 2022. The proportion of females was higher in the osteoporosis group (83.1%) compared to the control group (54.2%). A larger proportion of subjects in the control group were aged between 55 and 65 years compared to the osteoporosis group (Fig. S1).

Demographic characteristics are represented in Table 1, which were included as confounding factors in subsequent analyses: age, sex, ethnic background, economic index (multiple deprivation index), body measurements (body mass index [BMI] and waist-to-hip ratio [W/H]), comorbidities (diabetes, hypertension, hyperlipidemia, subacromial impingement syndrome [SIS], and subacromial bursitis [SBS]), activity frequency (moderate and vigorous activity days per week), lifestyle factors (smoking and alcohol intake frequency), and medication use (regular intake of vitamin D or calcium).

**Table 1:** Demographic and comorbidity characteristics of study cohort in the present study

Groups Characteristics	Missing (Proportion)	Overall		Osteoporosis		Control		*P*
		Mean ± SD Count	Mid (Min–Max) Percentage	Mean ± SD Count	Mid (Min–Max) Percentage	Mean ± SD Count	Mid (Min–Max) Percentage	
Samplesize		457 871		23 362		434 509		
Age at recruitment	3 (0.0%)	56.03 ± 8.06	57 (37–73)	60.81 ± 6.33	62 (40-71)	55.77 ± 8.07	57 (37–73)	<2.20e-16
Age at the recent version	3 (0.0%)	69.79 ± 8.07		74.58 ± 6.35		69.53 ± 8.08		<2.20e-16
Sex								<2.20e-16
Femal		255 009	55.7%	19 413	83.1%	235 596	54.2%	
Male		202 862	44.3%	3 949	16.9%	198 913	45.8%	
BMI	2 500 (0.5%)	27.34 ± 4.73		26.14 ± 4.81		27.41 ± 4.71		<2.20e-16
Waist/hip ratio	1 877 (0.4%)	0.87 ± 0.09	0.87 (0.20–2.97)	0.84 ± 0.08	0.83 (0.58-1.35)	0.87 ± 0.09	0.87 (0.20–2.97)	<2.20e-16
Ethnicity	1 677 (0.4%)							
White		429 956	93.9%	22 385	95.8%	407 571	93.8%	<2.20e-16
Asian		10 770	2.4%	432	1.8%	10 338	2.4%	2.15E-07
Black		7 593	1.7%	164	0.7%	7 429	1.7%	
Mixed		2 783	0.6%	93	0.4%	2 690	0.6%	2.78E-05
Others		6 769	1.5%	288	1.2%	6 481	1.5%	1.55E-03
Related comorbidities								
Diabetes		43 376	9.5%	2 738	11.7%	40 638	9.4%	<2.20e-16
Hypertension		146 279	31.9%	10 750	46.0%	135 529	31.2%	<2.20e-16
Hyperlipidemia		79 366	17.3%	6 275	26.9%	73 091	16.8%	<2.20e-16
Subacromial impingement syndrome		9 435	2.1%	679	2.9%	8 756	2.0%	<2.20e-16
Subacromial Bursitis		2 643	0.6%	200	0.9%	2 443	0.6%	9.98E-09
Smoking frequency	34 719 (7.6%)							3.00E-09
Most or all days		106 154	23.2%	5 550	23.8%	100 604	23.2%	
Occasionally		60 305	13.2%	3 054	13.1%	57 251	13.2%	
Once or twice		68 821	15.0%	3 168	13.6%	65 653	15.1%	
Never		187 872	41.0%	9 714	41.6%	178 158	41.0%	
Alcohol intake frequency	1 331 (2.9%)							<2.20e-16
Daily or almost daily		91 638	20.0%	4 196	18.0%	87 442	20.1%	
3–4 times/week		106 941	23.4%	4 583	19.6%	102 358	23.6%	
1–2 times/week		119 006	26.0%	5 540	23.7%	113 466	26.1%	
1–3 times/month		51 552	11.3%	2 594	11.1%	48 958	11.3%	
Special occasions		51 944	11.3%	3 598	15.4%	48 346	11.1%	
Never		35 459	7.7%	2 758	11.8%	32 701	7.5%	
Moderate physical activity	23 447 (5.1%)							<2.20e-16
0 day/week		54 228	11.8%	2 872	12.3%	51 356	11.8%	
1–2 day/week		99 957	21.8%	4 468	19.1%	95 489	22.0%	
3–5 day/week		174 743	38.2%	8 299	35.5%	165 444	38.1%	
6–7 day/week		105 496	23.0%	5 957	25.5%	99 539	22.9%	
Vigorous physical activity	23 549 (5.1%)							<2.20e-16
0 day/week		158 904	34.7%	9 655	41.3%	149 249	34.3%	
1–2 day/week		132 196	28.9%	5 952	25.5%	126 244	29.1%	
3–5 day/week		119 789	26.2%	4 880	20.9%	114 909	26.4%	
6–7 day/week		24 005	5.2%	1 161	5.0%	22 844	5.3%	
Multiple deprivation Index	11 526 (2.5%)	17.07 ± 13.86	12.55 (0.61–90.05)	17.85 ± 14.43	13.06 (0.61–88.24)	17.03 ± 13.83	12.52 (0.61–90.05)	<2.20e-16
Vitamine D supplementation	6 349 (1.4%)							<2.20e-16
Vitamin D		18 119	4.0%	2 573	11.0%	15 546	3.6%	
Multivitamin		93 397	20.4%	4 814	20.6%	88 583	20.4%	
Calcium supplementation	5 396 (1.2%)	31 610	6.9%	5 742	24.6%	25 868	6.0%	<2.20e-16

Compared to the control group, patients with osteoporosis exhibited significantly higher rates of comorbidity, including diabetes (11.7% vs. 9.4%), hypertension (46.0% vs. 31.2%), hyperlipidemia (26.9% vs. 16.8%), SIS (2.9% vs. 2.0%), and SBS (0.9% vs. 0.6%). The number of subjects reporting regular vitamin D intake in the osteoporosis group was significantly higher than that in the control group (11.0% vs. 3.6%), while those taking multivitamins were close.

### Cross-sectional prevalence of RCTs in subjects with osteoporosis

**Fig. 3:**
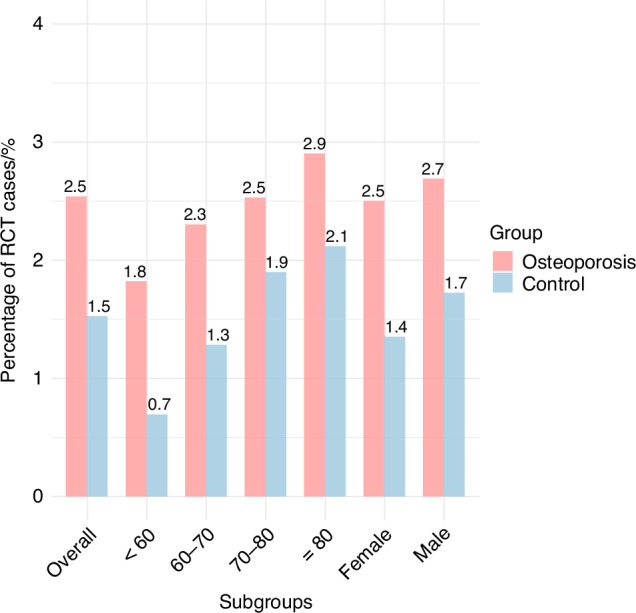
Comparison of RCT prevalence between the osteoporosis and control groups. The height of each bar represents the percentage of RCT cases within the osteoporosis (red) and control (blue) groups in the overall cohort, as well as in subgroups stratified by age and sex. Exact values are labeled above each bar

According to diagnoses from primary and inpatient health care systems, 23 362 subjects diagnosed with osteoporosis were classified in the osteoporosis group, while the other 434 509 were assigned in the control group. The prevalence of RCTs was significantly higher in the osteoporosis group (604/23,362; 2.6%) compared to controls (6 700/434 509; 1.5%). Subgroup analyses stratified by age (in 10-year intervals) and sex consistently demonstrated that the prevalence of RCTs among subjects with osteoporosis remained significantly higher than in controls across all subgroups (Fig. 3 and Table S1).

### Cross-sectional association between osteoporosis and RCT risk

Osteoporosis was significantly associated with increased RCT risk in both univariate and multivariate logistic regression models. Specifically, in the univariate logistic model, patients with osteoporosis had a significantly higher risk of developing RCTs (crude OR [95% CI]: 1.69 [1.56–1.84], *P* = 1.49E-34). Pooling results from imputed dataset, bidirectional stepwise multivariate logistic regression models revealed that osteoporosis was independently associated with RCTs (adjusted OR [95% CI]: 1.38 [1.25–1.52], *P* = 1.66E-10) after adjusting for confounding factors described above. Other significant predictors for RCT risks included male sex, higher age, non-White ethnicity, increased BMI and W/H, presence of hypertension, dyslipidemia, SIS and SBS, smoking frequency, and frequencies of moderate and vigorous physical activities (Table 2). Sensitivity analysis using unimputed data in the multivariate model confirmed consistent results (Table S2).

**Table 2:** Logistic regression analysis on osteoporosis and RCTs

Model	Variables	OR (95% CI)	*P*-value
Univariate model	Osteoporosis	1.69 (1.56–1.84)	1.49E-34
Multivariate model	Osteoporosis	1.38 (1.25–1.52)	1.66E-10
	Age	1.04 (1.04–1.05)	1.15E-97
	Sex (Female)	0.87 (0.81–0.94)	2.72E-04
	Ethnic backgroud (non-White)	1.41 (1.27–1.57)	9.81E-11
	W/H ratio	1.71 (1.10–2.66)	1.78E-02
	BMI	1.03 (1.03–1.04)	8.24E-27
	Hypertension	1.35 (1.28–1.43)	5.29E-24
	Hyperlipidemia	1.24 (1.16–1.31)	2.19E-11
	Impingement syndrome	47.07 (44.48–49.80)	<1E-300
	Bursitis	5.38 (4.78–6.06)	1.24E-169
	Smoking	1.06 (1.01–1.12)	1.91E-02
	Moderate physical activity	1.14 (1.07–1.23)	0.0002
	Vigorous physical activity	1.17 (1.07–1.27)	3.51E-04
	Vitamin supplementation		
	Multivitamin	1.09 (1.02–1.16)	0.0087
	Vitamin D	1.05 (0.93–1.20)	0.4153

*OR* odds ratio, *CI* confidence interval, *W/H ratio* waist to hip ratio, *BMI* body mass index

### Subgroup cross-sectional analyses on association between osteoporosis and RCT risk

To account for the potential effect of age, the cross-sectional cohort was stratified into younger (<70 years; 4 626 with osteoporosis vs. 202 664 controls) and older (≥70 years; 18 736 with osteoporosis vs. 231 842 controls) groups. A significant association between osteoporosis and RCTs was identified in both age groups in univariate (younger: OR [95% CI]: 2.05 [1.66–2.50], *P* = 5.01E-12; older: OR [95% CI]: 1.38 [1.26–1.51], *P* = 1.15E-11) and multivariate models (younger: adjusted OR [95% CI]: 1.65 [1.30–2.09], *P* = 3.92E-05; older: adjusted OR [95% CI]: 1.20 [1.08–1.34], *P* = 6.99E-07).

To account for the potential effect of sex, the cross-sectional subset was also stratified into male (3 949 with osteoporosis vs. 198 913 controls) and female (19 413 with osteoporosis vs. 235 596 controls) groups. Univariate logistic regression identified significant associations between osteoporosis and RCTs in both sexes (males: crude OR [95% CI]: 1.60 [1.32–1.94], *P* = 1.68E-06; females: crude OR [95% CI]: 1.90 [1.72–2.09], *P* = 4.74E-39). After adjusting for confounding factors, the association remained significant in females (adjusted OR [95% CI]: 1.43 [1.28–1.60], *P* = 5.44E-10), but became nonsignificant in males (*P* = 0.199 4).

### Longitudinal association between osteoporotic status and RCT risk

A subset of 262 931 subjects meeting inclusion and exclusion criteria was selected for longitudinal analysis with a follow-up time of 11 years. According to the criteria set by T-score reference value, this subset was grouped into 6 588 cases with osteoporosis, 70 862 cases with osteopenia, and 187 569 controls. As of October 2022, 126 (1.9%) subjects in the osteoporosis group, 780 (1.1%) in the osteopenia group, and 2088 (1.1%) in the control group had been diagnosed with RCTs. The detailed demographic characteristics of the subjects in the longitudinal subset were represented in Table S3.

**Fig. 4:**
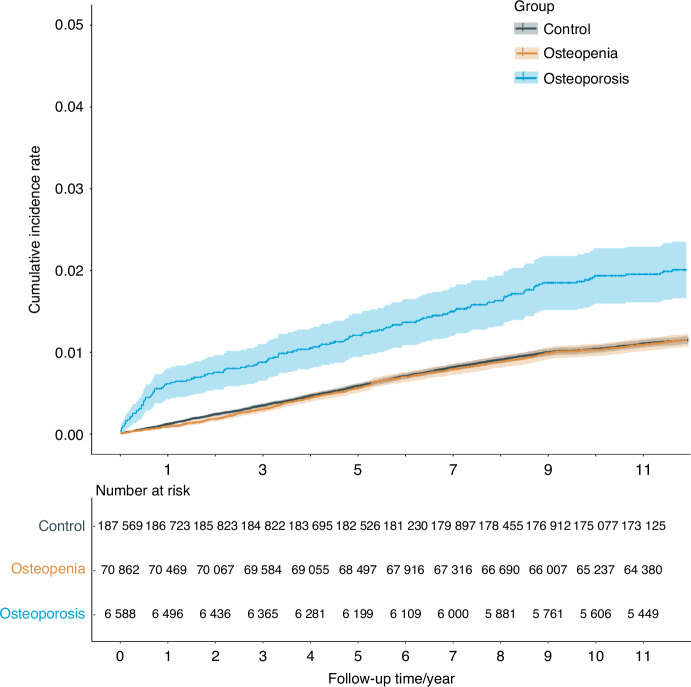
Cumulative risk curves for RCT incidence across groups. Survival curves depict the cumulative risk of rotator cuff tear prevalence in osteoporosis(blue), and osteopenia (brown) compared to controls (gray) during 11 years of follow-up. Numbers in the table under the curves indicates the number of subjects in each group at each time point

Cumulative risk curves (Fig. 4) illustrate the cumulative incidence of RCTs over a 11-year follow-up period across three groups. The risk of RCTs during the follow-up period remained significantly higher in the osteoporosis group compared to the other two groups, highlighting the substantial impact of osteoporosis (Log-rank test, *P* < 0.000 1), whereas no significant difference was observed between the osteopenia and control groups. The univariate model revealed that patients with osteoporosis had a significantly higher risk of developing RCTs compared to controls (crude HR [95% CI]: 1.79 [1.49–2.14], *P* = 2.68E-10) during the 11-year follow-up period, whereas no significant association was observed in patients with osteopenia. Bi-directional stepwise multivariate Cox regression modeling demonstrated that osteoporosis was independently associated with a significantly increased hazard of RCTs (pooled adjusted HR [95% CI]: 1.56 [1.30–1.87], *P* = 2.17E-06), whereas osteopenia did not show statistical significance. Additional significant predictors included increasing age, non-White ethnicity, higher BMI, W/H, and frequencies of moderate and vigorous activities, comorbidities of hypertension, hyperlipidemia, SIS and SBS. Sensitivity analysis using unimputed data in the multivariate model confirmed consistent results (Table S4).

As shown in Table 3, a simplified model generated through a bi-direction stepwise selection based on the lowest Akaike information criterion (AIC) identified osteoporosis as an independent predictor that optimized the model (likelihood ratio test [LRT], *P* < 2.2E-16). Furthermore, comparison of chi-square statistics between the full model and nested models excluding osteoporosis across each imputed dataset revealed that inclusion of osteoporosis significantly improved model fit (all *P*-values < 0.000 1). The optimal AIC and significant LRT confirmed that incorporating osteoporosis enhanced the model’s explanatory power, underscoring its independent predictive value in RCT development.

**Table 3:** Cox proportional hazard regression models on osteoporosis and RCTs

Model	Variable	HR (95% CI)	*P*-value
Univariate model	Osteoporotic condition		
	Osteopenia	1.00 (0.92–1.08)	0.916 0
	Osteoporosis	1.79 (1.49–2.14)	2.33E-10
Multivariate model	Osteoporotic condition		
	Osteopenia	1.06 (0.97–1.15)	0.191 6
	Osteoporosis	1.56 (1.29–1.87)	2.50E-06
	Age	1.03 (1.03–1.04)	2.09E-28
	Ethnic backgroud (non-White)	1.36 (1.14–1.62)	0.000 6
	BMI	1.03 (1.02–1.03)	5.67E-10
	W/H ratio	1.98 (1.25–3.13)	0.003 5
	Hypertension	1.36 (1.25–1.47)	3.21E-13
	Hyperlipidemia	1.20 (1.10–1.31)	2.66E-05
	Impingement syndrome	37.01 (34.18 - 40.07)	<1E-300
	Bursitis	2.84 (2.52–3.20)	8.05E-64
	Moderate physical activity	1.17 (1.05–1.29)	0.004 3
	Vigorous physical activity	1.10 (0.98–1.24)	0.098 7
	Vitamin supplementation		
	Multivitamin	1.08 (0.99–1.19)	0.075 8
	Vitamin D	0.91 (0.74–1.12)	0.379 4

*HR* hazard ratio, *CI* confidence interval, *W/H* ratio waist to hip ratio, *BMI* body mass index

Receiver operating characteristic (ROC) curve analysis revealed a slight increase in the area under the curves (AUC) across all imputed dataset, from 81.7% (81.6%–81.8%) in the nested model to 81.8% (81.7%–81.9%) in the full model (DeLong’s test *P* values = 0.079 4–0.105 1), suggesting a nominally improved predictive performance when including osteoporosis (Fig. S2). Although the increase in AUC was modest, it underscores the incremental value of incorporating osteoporosis into predictive models for RCT incidence.

### Subgroup longitudinal analyses on association between osteoporosis and RCT risk

Consistent with previously conducted cross-sectional analyses, subjects included in the longitudinal cohort were stratified into age-based subgroups: ≤70 years (1 321 with osteoporosis, 25 874 with osteopenia, and 87 125 controls) and >70 years (5 267 with osteoporosis, 44 988 with osteopenia, and 100 444 controls). The association between osteoporosis and RCTs remained significant in both age subgroups after adjusting for confounding factors (age ≤70 years: HR [95% CI] = 1.91 [1.23–2.96]; age >70 years: HR [95% CI] = 1.47 [1.20–1.81]).

Subjects were then stratified into sex-based subgroups: males (1 697 osteoporosis vs. 25 147 vs. osteopenia vs. 96 187 controls) and females (4 891 osteoporosis vs. 45 715 vs. osteopenia vs. 91 382 controls). In univariate and multivariate Cox regression analysis, osteopenia and osteoporosis were significantly associated with increased RCT risk in females (adjusted HR [95% CI]: 1.12 (1.01–1.26) for osteopenia, and 1.66 (1.34–2.05) for osteoporosis, *P* < 0.05), but not in males, where no significant association was found in either univariate or multivariate Cox models (*P* > 0.05).

### Sex-interaction underlying osteoporosis and RCT risk

**Fig. 5:**
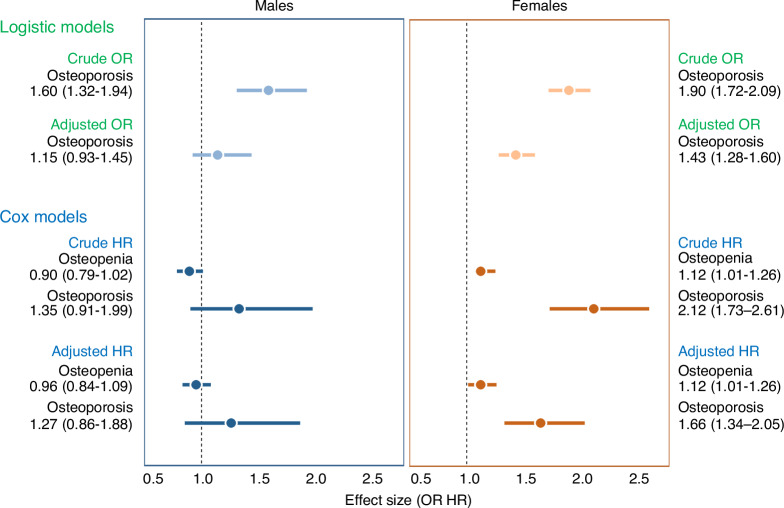
Forest plots of association between osteoporosis and RCTs in sex-stratified analysis. The forest plot displays the crude and adjusted ORs from logistic regression models and HRs from Cox models for males (left) and females (right). The horizontal lines represent 95% confidence intervals

A forest plot (Fig. 5) was generated to illustrate the effects of osteoporosis on RCTs in sex-stratified analyses across both cross-sectional and longitudinal subsets. In the multivariate models, the association between osteoporosis and RCT risk did not reach statistical significance among males. In contrast, females exhibited a significantly higher hazard associated with osteoporosis compared to males. Given these sex-specific differences, we further conducted interaction analyses by including a sex × osteoporosis interaction term in the multivariate Cox models.

Both osteopenia and osteoporosis were independently associated with increased RCT risk. Specifically, osteopenia was linked to a 14% increased hazard (*P* = 0.019 0), while osteoporosis was associated with a 71% increase (*P* = 7.75E-07). A significant sex × osteopenia interaction was detected, indicating a weaker effect in males (HR [95% CI] = 0.84 [0.47–1.00], *P* = 0.046), suggesting that osteopenia contributes less to RCT risk among men. Sex × osteoporosis interaction was not statistically significant (HR [95% CI] = 0.74 [0.48–1.15], *P* = 0.183 8), but a trend toward a reduced effect in males was observed.

**Fig. 6:**
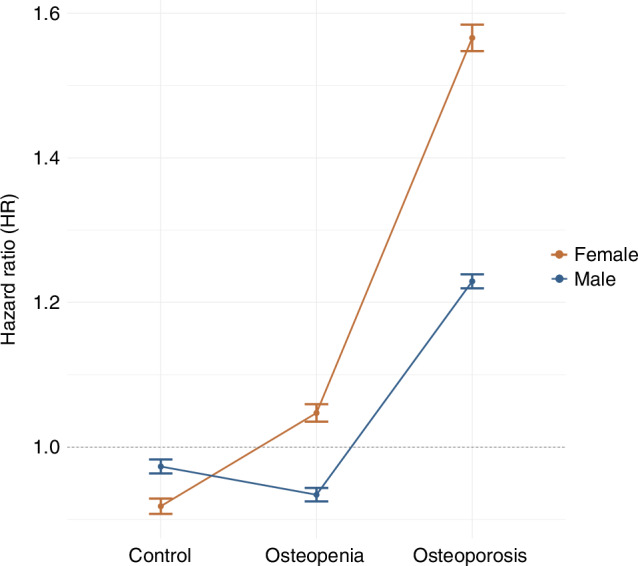
Predicted risk effect of RCTs by osteoporotic status and sex. Predicted HRs for RCTs are shown across control, osteopenia, and osteoporosis groups, stratified by sex (female in orange, male in blue). Error bars represent 95% confidence intervals. The dotted line at HR = 1.0 indicates the reference level (control group)

To illustrate the interaction effect of sex, six hypothetical subgroups were constructed by combining osteoporotic condition (normal, osteopenia, osteoporosis) and sex (male, female). Predicted hazard ratios from the stepwise multivariate Cox model were visualized in Fig. 6, showing diverging trends of hazard ratios. Specifically, females exhibited an increasing RCT risk across osteopenia and osteoporosis compared to controls. In males, the trend was not significant, but those with osteoporosis had a notably higher risk than those with osteopenia or controls. These findings suggest a sex-specific pattern in the association between osteoporosis and RCT risk, with a cumulative effect observed in females. Notably, in the control group, the predicted hazard for RCT in males were higher than females. This is aligned with the finding in the cross-sectional analyses, where the RCT prevalence was higher in males compared to females across the overall cohort and age subgroups (Fig. S3), and male sex was an independent risk factor for RCT risk in the multivariate logistic model.

### Propensity score-based analyses on causality of osteoporosis on RCTs

Propensity score for each subject in the cross-sectional and longitudinal subsets were calculated using logistic regression models to balance the confounding factors associated with osteoporosis. Based on 1:5 matching, 116 810 and 32 940 controls were successfully matched to 23 362 and 6 588 osteoporosis patients for cross-sectional analysis and longitudinal analysis, respectively. Sensitivity analysis was conducted in alternative parameters using 1:3 and 1:1 matching. In the balance tests, standardized mean differences (SMDs) for all covariates were reduced to below 0.1 after matching, with adjusted mean differences close to zero, indicating satisfactory covariate balance between groups. These were further illustrated in the Love plots (Fig. S4), which visually demonstrates that all post-matching SMDs fall well within the accepted threshold for balance.

Using the matched datasets, osteoporosis was significantly associated with RCT risk in the cross-sectional analysis (pooled OR [95% CI] = 1.36 [1.22–1.51], *P* = 2.15E-08). Sensitivity analyses using alternative matching ratios (1:3 and 1:1 matching) yielded consistent results, with pooled ORs (95% CI) of 1.36 (1.12–1.54, *p* = 2.36E-06) and 1.34 (1.19–1.50, *P* = 0.000 4), respectively. In the longitudinal analysis using the 1:5 matched dataset, osteoporosis remained significantly associated with increased RCT risk (pooled HR [95% CI] = 1.46 [1.16–1.84], *P* = 0.001 9). This association was further supported by sensitivity analyses using 1:3 (HR = 1.43 [1.10–1.86], *P* = 0.007 3) and 1:1 (HR = 1.49 [1.05–2.12], *P* = 0.026 7) matching ratios. While smaller sample sizes slightly weakened statistical significance, all PSM-based analyses consistently supported a robust causal effect of osteoporosis on RCT risk.

Additionally, propensity scores were used to perform weighted logistic and Cox regression analyses, further confirming the significant association. The weighted OR (95% CI) for the cross-sectional analysis was 1.51 (1.46–1.56), and the weighted HR (95% CI) for the longitudinal analysis was 1.79 (1.49–2.15), both with *P* < 0.000 1.

**Fig. 7:**
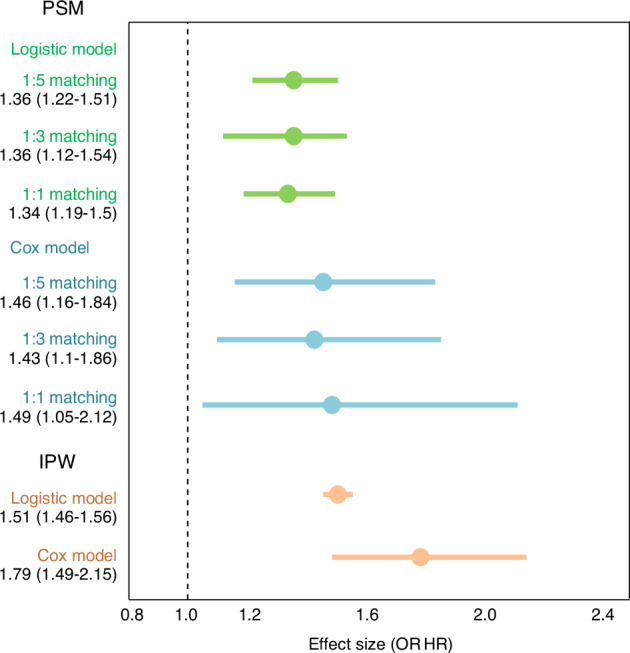
Forest plots of association between osteoporosis and RCTs in PS-based analyses. Forest plot showing the effect sizes (OR or HR) and 95% CI from different analytical models. PSM was conducted with 1:5, 1:3, and 1:1 matching ratios, analyzed separately using logistic regression and Cox proportional hazards models. IPW analysis results are presented for both logistic and Cox models

Results from the PS-based analyses are visualized in the forest plot (Fig. 7).

### Machine learning-based analyses on causality of osteoporosis on RCTs

CRF and SRF models were respectively applied to estimate the average treatment effect (ATE) of osteoporosis on RCT risks in cross-sectional (23 362 cases vs. 434 509 controls) and longitudinal subjects (6 588 cases vs. 187 569 controls), respectively. Osteoporosis was designated as the treatment variable (osteoporosis = 1, control = 0). Covariates were included in the models to control for potential confounding factors. In the CRF model, a significant causal effect of osteoporosis on the risk of RCTs was identified (ATE ± SE = 0.0075 ± 0.0011, *P* = 2.40E-12), suggesting that individuals with osteoporosis have a 0.75% higher probability of developing RCTs compared to controls on average. In the SRF model, osteoporosis was causally associated with a decreased probability of remaining disease-free from RCTs over a 11 years of follow-up (ATE = -0.007 2 ± 0.002, *P* = 0.000 3), indicating a 0.72% of increased probability of developing RCTs over time in individuals with osteoporosis.

For sensitivity analyses, 1:5 matched subsets from the cross-sectional and longitudinal cohorts were reanalyzed using CRF and SRF models, respectively. Additionally, causal effects were estimated over additional follow-up periods of 5 and 3 years, further confirming the robustness of the causal relationship between osteoporosis and increased RCT risk. Detailed results were represented in Table 4.

**Table 4:** Average treatment effect of osteoporosis on RCTs

Model	Variable	ATE	SE	*p*
Causal random forest	Overall cohort	0.007 5	0.001 1	2.4E-12
	1:5 matching subset	0.006 0	0.001 1	6.09E-08
Survival random forest	Overall cohort	−0.007 2	0.002 0	0.000 3
	11 years			
	1:5 matching subset			
	11 years	−0.007 9	0.002 0	6.09E-05
	5 years	−0.006 4	0.001 6	6.29E-05
	3 years	−0.005 0	0.001 3	4.58E-05

*ATE* average treatment effect, *SE* standard error

### LDSC analysis evaluating genetic correlation between osteoporosis and RCTs

Based on LDSC analysis, the heritability of osteoporosis risk was estimated at 0.008 in the Finn Biobank cohort, while bone mineral density (BMD) at different sites heritability varied between 0.072 and 0.253 in the Genetic Factors for Osteoporosis Consortium (GEFOS) cohorts. The heritability of RCTs was estimated at 0.008 and 0.014 in the UK Biobank and (Kaiser Permanente Northern California KPNC cohorts), respectively. However, paired LDSC analysis did not demonstrate a significant genetic correlation between osteoporosis-related traits and RCTs (*P* > 0.05), among which only the femoral neck BMD and RCTs of the UK biobank showed a nominal significance in genetic correlation (*P* = 0.070 3). These results suggested that shared mechanisms between osteoporosis and RCTs in genetics are likely minor. Detailed results were presented in Table S5.

### Colocalization analysis identifying shared genetic locus for osteoporosis and RCTs

Although the overall genetic correlation is weak between osteoporosis and RCTs, but colocalization analysis successfully identified several potential loci for BMD and RCTs, suggesting a shared genetic mechanism.

In the colocalization analysis, four loci (*WNT2B* rs3737136, *CD109* rs9442952, *KLHL42* rs11049197, and *CFH* rs1576340) exhibited strong posterior probabilities (*PP*_*H4*_ > 70%), and two loci (DNM3 rs12401515 and *PKDCC* rs12996954) showed moderate posterior probabilities (*PP*_*H4*_ = 50% to 70%), indicating shared loci between osteoporosis-related traits and RCTs. Detailed results of the primary colocalization analysis were represented in Table 5 and Fig. S5.

**Table 5:** Colocalization analysis on osteoporosis-related traits and RCTs

Colocalization analysis			Osteoporosis-related traits			RCTs			External replication for RCTs		
Locus	nSNP	PP(H4)	Trait	Beta (Se)	*P*-value	Source	Beta (Se)	*P*-value	Source	Beta (Se)	*P*-value
rs3737136	1 815	91.7%	Skull BMD	−0.05 (0.01)	1.10E-07	UKB	−0.11 (0.02)	1.44E-05	KPNC	0.01 (0.02)	0.595 1
rs9442952	2 822	70.8%	Skull BMD	−0.03 (0.01)	6.40E-06	UKB	0.07 (0.02)	2.58E-04	KPNC	−0.10 (0.05)	0.040 1
rs11049197	2 731	89.8%	Femoral neck BMD	−0.05 (0.01)	3.29E-06	UKB	0.12 (0.03)	5.67E-06	KPNC	−0.04 (0.02)	0.091 3
rs12401515	1 507	57.9%	Femoral neck BMD	0.03 (0.01	0.0251	KPNC	0.12 (0.03)	1.69E-05	UKB	−0.04 (0.02	0.133 4
rs12996954	3 541	65.0%	Forearm BMD	0.08 (0.02)	1.77E-05	UKB	−0.09 (0.02)	2.05E-04	KPNC	−0.07 (0.02)	9.28E-04
rs1576340	1 828	76.8%	Forearm BMD	0.03 (0.01	0.0041	KPNC	0.07 (0.02)	1.15E-04	UKB	0.005 (0.02	0.840 3

*PP(H4)* posterior probability (Hypothesis 4), *Se* standard error, *BMD* bone mineral density

**Fig. 8:**
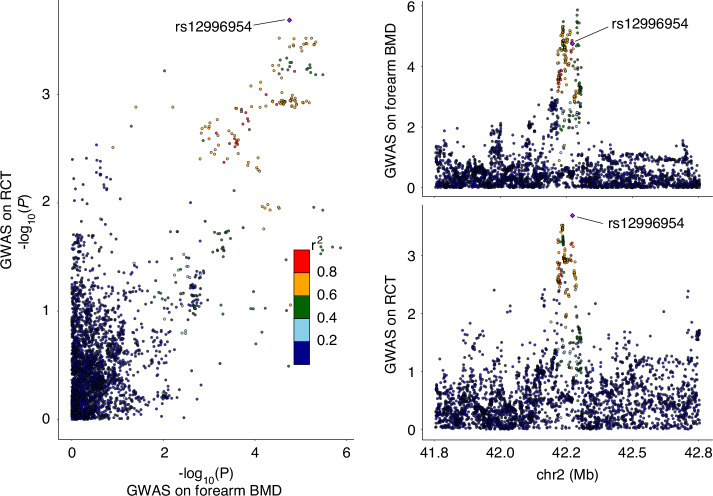
Shared genetic locus for forearm BMD and RCTs identified through colocalization analysis. The lead SNP rs12996954 is marked with a purple diamond, indicating the most significant colocalization among the SNPs of the test region. The plot on the right illustrates causal variation for both traits from the same locus, while −log *P* values for the two traits from a single locus are presented on the left

**Fig. 9:**
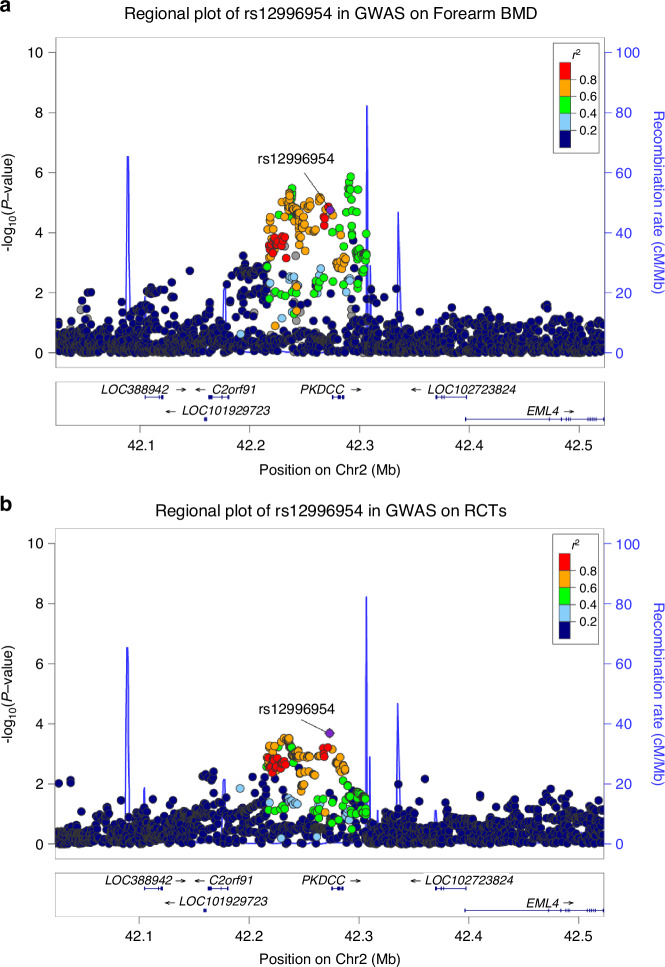
Regional plots of *PKDCC* rs12996954 in GWASs. The plots illustrate the associations of single within a 250 kb region centered on the colocalized SNPs. The X-axis indicates the chromosomal position of the SNPs, while the Y-axis represents the log-transformed *P*-values (−log_10_). Each scatter point represents a SNP, whose color indicating the linkage disequilibrium (*r*²) with rs12996954. **a** GWAS of forearm bone mineral density. **b** GWAS of rotator cuff tears

Focusing on the six loci, a secondary colocalization analysis with soft thresholds were conducted to explore the evidence in the replication of their associations with other osteoporosis-related traits and RCTs of the other cohort. Notably, rs12996954 was identified to be shared between forearm BMD and RCTs of the UK Biobank cohort (Fig. 8). The secondary colocalization analysis revealed its colocalization within femoral neck BMD and RCTs of the UK Biobank cohort (*PP*_*H4*_ = 80.3%). Although the secondary analysis did not yield the colocalization of rs12996954 within osteoporosis-related traits and RCTs of the KPNC cohort, a cross-referencing on GWAS summary statistics of the KPNC study identified the significant association of rs12996954 with RCTs in the KPNC cohort (*P* = 0.000 9 < 0.05/6). Fine mapping indicated this locus was mapped to the upstream region of the *PKDCC* gene (Fig. 9). The correlation trends of this locus with BMD or RCTs were consistent across studies.

Secondary analysis further confirmed the potential association of *WNT2B* rs3737136, *CFH* rs12401515, and *DNM3* rs1576340 with osteoporosis-related traits, but their association with RCTs was not replicated in the present study. Detailed results of the secondary colocalization analysis were represented in Table 6.

**Table 6:** Secondary colocalization analysis on candidate shared loci on osteoporosis-related traits

Loci	Trait	Colocalization analysis		Osteoporosis-related traits	
		nSNP	PP(H4)	Beta (Se)	*P*-value
rs3737136 (G > A)	Osteoporosis	2 216	43.5%	0.02 (0.02)	0.398 2
	Skull BMD	^a^			
	Femoral neck BMD	2 099	97.4%	0.04 (0.01)	4.03E-05
	Forearm BMD	2 143	13.2%	0.02 (0.02)	0.271 4
	Spine BMD	2 099	81.6%	0.03 (0.01)	0.001 8
rs9442952 (G > A)	Osteoporosis	3 292	7.6%	0.003 (0.02)	0.861 4
	Skull BMD	^a^			
	Femoral neck BMD	3 078	8.6%	0.000 03 (0.01)	0.997 2
	Forearm BMD	3 131	9.7%	-0.02 (0.02)	0.345 9
	Spine BMD	3 078	6.2%	-0.01 (0.01)	0.580 3
rs11049197 (A > G)	Osteoporosis	2 898	15.6%	-0.02 (0.02)	0.288 7
	Skull BMD	2 604	1.7%	-0.002 1 (0.01)	0.822 9
	Femoral neck BMD	^a^			
	Forearm BMD	2 819	10.7%	-0.01 (0.02)	0.594 0
	Spine BMD	2 780	36.0%	-0.01 (0.01)	0.380 3
rs12401515 (A > G)	Osteoporosis	2 002	13.0%	-0.02 (0.02)	0.341 6
	Skull BMD	1 601	5.3%	0.04 (0.01)	0.001 8
	Femoral neck BMD	^a^			
	Forearm BMD	1 830	24.7%	0.00 (0.02)	0.994 3
	Spine BMD	1 799	67.6%	0.026 9 (0.01)	0.025 1
rs12996954 (G > A)	Osteoporosis	3 536	8.1%	-0.01 (0.02)	0.480 4
	Skull BMD	3 064	34.1%	0.02 (0.01)	0.025 2
	Femoral neck BMD	3 459	80.3%	0.04 (0.01)	2.04E-05
	Forearm BMD	^a^			
	Spine BMD	3 458	5.4%	-0.000 1 (0.01)	0.992 7
rs1576340 (G > T)	Osteoporosis	1 309	7.9%	-0.01 (0.02)	0.434 4
	Skull BMD	1 164	5.0%	0.00 (0.01)	0.639 3
	Femoral neck BMD	1 188	37.9%	0.02 (0.01)	0.009 0
	Forearm BMD	^a^			
	Spine BMD	1 187	60.0%	0.031 1 (0.01)	0.004 1

Secondary colocalization analysis using a loose threshold of prior probability (*P* < 0.05 for each traits, *P* < 0.001 for both traits)

*nSNP* number of SNPs in the locus, *PP* the posterior probability of each hypothesis, *Se* standard error

^a^Identified in primary analysis

### Functional annotations of candidate loci for osteoporosis and RCT

The annotations for the six identified loci based on public databases are presented in Table S6. Focusing on *PKDCC* rs12996954, as recorded in GTEx database, it was identified as both an expression quantitative trait locus (eQTL) and a splicing quantitative trait locus (sQTL) across various tissue and cell types, such as esophagus muscularis, skeletal muscle and cultured fibroblasts, where it regulates the downregulation of *PKDCC* expressions. Additionally, *PKDCC* is considered a skeletal muscle-enriched gene, suggesting that the effect of eQTL/sQTL may be more pronounced in skeletal muscles compared to other tissues or system. We analyzed the genomic region centered by the *PKDCC* rs12996954 using RegulomeDB. This region contains 84 regulatory peaks, suggesting the presence of potential regulatory elements such as transcription factor binding sites or enhancers. The region was ranked “2A”, indicating a moderate likelihood of regulatory function, based on available evidence. The score of 0.68 reflects moderate confidence in its regulatory role, though further experimental validation is needed.

## Discussion

In this study, we conducted a prospective cohort analysis using data from the UK Biobank to investigate the association between osteoporosis and RCTs. Osteoporosis was significantly associated with increased RCT risk, particularly among females. Multiple PS-based and ML-based causal inference approaches further supported a causal relationship. Colocalization analysis identified six candidate loci shared between BMD and RCTs, with PKDCC rs12996954 showing strong external replication. Our findings not only provide evidence for osteoporosis as a causal factor for RCTs, but also provide novel insights into the shared genetic and molecular mechanisms that may partially explain the clinical correlation between these two conditions.

Although clinical observations have long suggested a link between osteoporosis and RCTs, causal inference has been hindered by confounding factors. Our prospective design, along with an 11-year follow-up, provides strong evidence for causality, with osteoporosis conferring a 1.56-fold increased risk of RCTs, consistent with findings from a cohort (1.79-fold increased risk).^7^ Prior studies have also reported associations between osteoporosis and RCT morbidity and healing.^8,9,17^

Notably, PS-based and ML-based analyses in our study present novel evidence on causality and underscore the significance of osteoporosis management in prevention of RCTs. Previous studies in patients^18^ and animal models^19,20^ have demonstrated the positive effect of osteoporosis treatments (e.g., zoledronic acid, alendronate, and abaloparatide) on tendon healing after RCR. However, whether these treatments can reduce the risk of developing RCTs remains unknown. Our preliminary analysis did not identify a protective effect of calcium or vitamin D supplementation in preventing RCTs. This may be attributed to the relatively mild anti-osteoporotic effects of these supplements or the influence of complex osteoporosis pathology.

Sex-stratified analysis revealed a higher RCT risk associated with osteoporosis in females compared to males (adjusted HRs for males vs. females: 1.27 vs. 1.64). This finding is consistent with estimates from a cohort study (adjusted HRs for males vs. females: 1.55 vs. 1.82).^7^ Interaction analysis further demonstrated that sex modifies the impact of osteoporosis on RCT risk. Additionally, sex-stratified analysis showed that RCT risk increased with the severity of osteoporotic status in females. Although the association between osteoporosis and RCTs in males was not statistically significant—potentially due to the smaller sample size in male cases of osteopenia and osteoporosis—the trends were consistent across sexes. Although hormone disorders are common causal factors for osteoporosis in both sexes, the significantly decreased estrogen levels following ovarian dysfunction accelerate osteoporosis progression in postmenopausal women.^21^ Estrogen deficiency in postmenopausal women, along with its exacerbation of osteoporosis, may contribute to greater RCT pathology and partly explain the heightened susceptibility to RCTs observed in females.

Notably, the overall incidence of RCTs in males was significantly higher than females (1.8% vs. 1.4%) in the cross-sectional cohort, consistent with previous studies.^22,23^ Male sex was identified as an independent risk factor for RCTs in the multivariate logistic regression analysis. This suggests that distinct sex-related mechanisms also contribute to RCT pathogenesis, highlighting the complexity of its etiology and the need for personalized therapeutic strategies.

Osteoporosis and RCTs are both complex and degenerative diseases that share common risk factors, such as aging-related degeneration, sex-related hormone differences, and environmental influences. Furthermore, they may share molecular mechanisms in their pathologies, including disorders in stem cell proliferation and differentiation,^24,25^ extracellular matrix (ECM) expression,^26,27^ or fibroblast growth factor signaling.^28,29^ Identifying these shared molecular mechanisms help to understand the strong clinical correlation observed in cohort studies and develop optimized therapeutic strategy.

Through colocalization analysis, we identified *PKDCC* rs12996954 (G>A) as a shared locus for BMD and RCTs. *PKDCC* encodes a secreted tyrosine-protein kinase that phosphorylates members of the matrix metallopeptidase (MMP) family (e.g. MMP1, MMP13, and MMP14),^30^ playing a critical role in the ECM degradation and remodeling, contributing to the regulation of musculoskeletal system developments. *PKDCC* rs12996954 was reported to be significantly associated with BMD in an independent GWAS.^31^ Congenital deficiency in *PKDCC* has been linked to disorders in skeletal system development,^32,33^ manifested by increased proliferative chondrocytes, widened proliferative bands, and delayed bone mineralization. These findings provide external validations to support the correlation of this locus with BMD and osteoporosis identified in our analysis.

Although the association between *PKDCC* rs12996954 and RCTs was only nominally significant in the UK Biobank cohort, it was replicated in the KPNC cohort. The observed colocalization of *PKDCC* rs12996954 for both BMD and RCTs, together with evidence of its colocalization between bone and muscle,^34^ supports a pleiotropic role for *PKDCC* in musculoskeletal health. The G allele of rs12996954 was associated with lower BMD and increased RCT risk and also acts as an expression quantitative trait locus located 2 kb upstream of *PKDCC*, where the G allele correlates with reduced *PKDCC* expression.

Mechanistically, this locus may contribute to RCTs through two potential mechanisms. First, collagens, the primary components of tendon fibers,^35^ may be regulated by members of the MMP family, with *PKDCC* playing a role in their phosphorylation. Second, the enthesis is a structure connecting tendon to bone, which histologically transitions from dense fibrous tendon to fibrocartilage, mineralized fibrocartilage, and bone.^33,36^ Given the role of *PKCDD* in the development and mineralization of bone tissue, this locus may affect the development and function of the rotator cuff enthesis and, consequently, the risk of RCTs. This may also be the potential mechanism of the positive effect of anti-osteoporosis on improving tendon healing in animal study.

This study has several limitations. First, RCT case identification was based on healthcare records, capturing only symptomatic individuals and potentially missing asymptomatic cases, similar to limitations observed in the original GWAS datasets. Future studies incorporating imaging data could enhance diagnostic precision. Second, although we included medication data for vitamin D and calcium supplementation, uncertainty regarding the vitamin D content of multivitamin supplements remained. Sensitivity analyses showed no consistent association between vitamin intake and RCT risk after adjusting for covariates and interactions. The potential for residual confounding persists, warranting future research with more precise exposure measurements. Third, although exercise frequency was adjusted for, the lack of detailed physical activity profiles limited our ability to fully account for biomechanical exposures. While the use of chronic injury diagnostic codes likely minimized the inclusion of acute trauma cases, this limitation should be addressed in future cohort designs. Finally, although we identified promising colocalized loci, functional validation is required to confirm their roles in the pathogenesis of osteoporosis and RCTs.

In conclusion, using data from the large UK Biobank cohort, we not only confirmed the association between osteoporosis and RCTs through correlation analysis but also provided evidence supporting a causal relationship using PS-based and ML-based inference methods. Additionally, colocalization analysis identified *PKDCC* rs12996954 as a shared locus for BMD and RCTs, suggesting a horizontal genetic correlation between the two conditions. These findings offer robust support for osteoporosis as a risk factor for RCTs and provide new insights into their shared pathology and potential therapeutic strategies.

## Materials and methods

### Study design

Firstly, correlation analyses between osteoporosis and RCTs were conducted using data from the UK Biobank (released by October 2022), a large-scale, prospective biomedical database with lifestyle, health, genetic information, and biological samples from 502 370 adults in England, Scotland, and Wales. Cross-sectional and longitudinal analyses were conducted to compared the prevalence of RCTs of individuals with osteoporosis to controls and assessed the impact of osteoporosis on RCT risk over an ~11-year follow-up period. In addition, multiple approaches, including PS-based methods (PSM and IPW) and ML-based models (CRF and SRF), were employed to establish causality between osteoporosis and RCTs. Finally, bioinformatics analyses, including LDSC analysis and colocalization analysis were performed to identify the genetic correlations between osteoporosis and RCTs, using GWAS summary statistics for osteoporosis risk and multisite BMD from the Finn Biobank and the GEFOS study, along with RCTs GWAS data from the UK Biobank and the KPNC cohorts.

### Study subjects screening and grouping

Participants were recruited from NHS registers between 2006 and 2010, with follow-up medical records linked from primary care, inpatient care, and death registries. Detailed descriptions of the UK Biobank study design and data collection methods are available in a previous publication elsewhere.^37^ The UK Biobank study had been conferred with ethics ratification (21/NW/0157) by the NHS North West Multicenter Research Ethics Committee, and all data used in this study were de-identified, eliminating the need for further ethical review. The most recent data collection concluded in October 2022, with release in 2023.

In the cross-sectional analysis, deceased subjects before October 2022 were excluded prior to analysis. Subjects with osteoporosis diagnosis were included in the case group, while those without an osteoporosis diagnosis served as controls. For the longitudinal analysis, subjects were included if they met either of the following criteria: (i) a heel BMD measurement obtained via ultrasound bone densitometry at the UK Biobank assessment center by 2011 or (ii) an osteoporosis diagnosis recorded in primary or inpatient healthcare records by 2011. Subjects were excluded if they met any of the following criteria: RCT diagnosis before 2011. Subjects with a heel BMD t-score below −2.5 or an osteoporosis diagnosis were included in the osteoporosis group. Subjects with a heel BMD t-score between −2.5 and −1 were included in the osteopenia group. Those with a heel BMD above −1 in 2011 and no osteoporosis diagnosis ever were assigned to the control group.

### Variable definition and data processing

Demographic, clinical, lifestyle, and comorbidity data were extracted using data extraction tool (“ukbconv.exe”) from the UK biobank according to specific fields. Osteoporosis and RCT diagnoses were identified using primary care (Read2/Read3) and inpatient (ICD-10) codes. A full list of diagnosis codes of comorbidities, filed and encoding of variables is provided in Supplementary Methods and Table S7. Missing data for these variables were all below 8% and were addressed using multiple imputation by chained equations (MICE), with five imputed datasets generated (*m* = 5). Density plots showing the imputation quality are presented in Fig. S6. Detailed variable definitions and missing imputation method are provided in the Supplementary Materials.

### Identification of independent association between osteoporosis and RCTs

In the cross-sectional analysis, a Chi-square test compared comorbidity rate of RCT in the osteoporosis group to the control group. Univariate and multivariate logistic regression models were employed to analyze the correlation between osteoporosis and RCT risk. In the longitudinal analysis, univariate and multivariate Cox proportional hazards regression were conducted to evaluate the independent effect of osteoporosis on the RCT risk over an 11-year follow-up period. A stepwise selection method based on AIC values was applied to establish parsimonious models and determine the role of osteoporosis as a predictor for RCTs. LRT and ROC curve compared the goodness-of-fit and the predictive effectiveness of the full Cox model to a nested model excluding osteoporosis. A cumulative risk curve illustrated the probability of RCT incidence over time, and a log-rank test assessed significance in RCT risk differences between the case and control groups. Additional subgroup analysis investigated heterogeneity by sex and age, applying Cox regression to test the association between osteoporosis and RCT within each subgroup. Results of imputation datasets were pooled by “Rubin’s Rules”.

### PS-based analysis on causality between osteoporosis and RCTs

PSM and IPW are statistical methods that use PSs to balance covariates between groups during model fitting. In the PSM method, a specific proportion of control subject is matched to each case based on their PSs, so as to minimize heterogeneity. In this study, PSs were estimated using multivariate logistic regression models for cross-sectional and longitudinal analyses, respectively. Osteoporosis cases were matched with controls using nearest PSs at a 1:5 ratio, and the matched samples were then used to fit the logistic and Cox regression models. Alternative ratio, 1:3 and 1:1, were applied to matched different subsets for sensitivity analyses.

For the IPW method, PSs are used to calculate weights, defined as the inverse probability of receiving the observed treatment, with osteoporosis cases weighted as $1-{PS}$, while controls weighted as $1/(1-{PS})$. These weights balance covariate distribution between the osteoporosis and control groups. Each subject was received a weight derived from their PSs, which was applied in the logistic and Cox regression models to evaluate the weighted effect of osteoporosis on RCT risk in the cross-sectional and longitudinal subsets, respectively.

### ML-based analysis on causality between osteoporosis and RCTs

In the CRF model for cross-sectional cohort and SRF model for longitudinal cohort, osteoporosis exposure was treated as a binary variable representing treatment assignment. RCT occurrence was modeled as a binary response variable in both models, while the SRF model also incorporating time to RCT as a continuous variable to capture both event status and time to event. Confounders were included as covariates to control for their potential impact on treatment estimation. The CRF and SRF models were both constructed with 2 000 trees, with each tree trained on 50% of the sample through bootstrap sampling. Sub-models for tuning consisted of 200 trees each, and 50 sub-models were used to optimize the overall model performance. An alpha parameter of 0.05 was set to control the maximum imbalance at each split, ensuring that each child node contained at least one failure or 5% of the sample size in the parent node. The ATE was employed to estimate the causal effect of osteoporosis on RCT incidence. Sensitivity analyses of SRF were conducted using alternative time horizons (3 years and 5 years), as well as in the 1:5 matching subset.

### Investigating genetic correlation between osteoporosis and RCTs

GWAS summary statistics of osteoporosis-related traits included a GWAS on osteoporosis risk by the Finn Biobank and multiple GWASs on BMD from the GEFOS consortium, covering lumbar spine, femoral neck, forearm, and skull. The GWAS summary statistics of RCTs were derived from two independent cohort studies aimed at identifying genetic variants for RCT risk, including the UK Biobank^38^ cohort and KPNC^39^ cohort. Dataset details of the data sources are provided in Table S8.

A LDSC analysis was conducted to calculate their heritability and the overall genetic correlations between each osteoporosis-related trait and RCTs based on the GWAS summary statistic datasets. Colocalization analysis was performed to identify shared genetic loci between osteoporosis-related traits and RCTs using GWAS summary statistics. A posterior probability (PP) was estimated by Bayesian method,^16^ where PP_H4_ ≥ 70% indicated strong evidence for colocalization, and 50%–70% indicated moderate evidence. A secondary analysis with relaxed thresholds (*P* < 0.05 for individual traits; *P* < 0.001 for both) was performed to assess colocalization at loci identified in the primary analysis.

Colocalized loci were functionally annotated using Ensembl, GTEx, and the Human Protein Atlas. Transcriptional regulatory potential was assessed using RegulomeDB. Details of colocalization analysis and genetic locus annotation are provided in the Supplementary Materials.

### Statistical analyses

Continuous variables are presented as mean ± standard deviation (SD), and categorical variables as count (percentage). A student’s *T* test and a chis-square test were used to test the difference of demographic and comorbidity characteristics across groups. Logistic regression model and Cox proportional hazards model were used to evaluate the association between osteoporosis and RCTs. OR and HR were used to describe the effect size of variables. Multiple imputation was conducted using “mice” (v3.17.0) package. PSM was conducted using “MatchThem” (v1.2.1) package. CRF and SRF analyses used “grf” (v2.3.2) package. LDSC and colocalization analysis were conducted with “ldscr” (v0.1.0) and “coloc” (v5.2.3) packages, respectively. Colocalized loci were visualized using “locuszoomr” (v0.3.0) package. Statistical significance was set at *P* < 0.05. All analyses and data visualization were performed using R software (v4.3.1).

## Supplementary information

Supplemental Materials

simulated dataset and R codes
