# Supplementary material for: Association between osteoporosis and rotator cuff tears: evidence from causal inference and colocalization analyses
Source: Bone Res. 2025 Aug 28;13:75. doi: 10.1038/s41413-025-00450-z (PMC12391355; doi:10.1038/s41413-025-00450-z)
Supplement: Supplementary file 1 — Supplemental Materials [file 41413_2025_450_MOESM1_ESM.pdf]

Supplemental materials

Supplemental methods

1. Variable definition

Diagnosis records for subjects with osteoporosis and RCTs were retrieved using Read2 and Read3 codes in primary health care and ICD-10 codes in inpatient health care systems (Field IDs: 41270, 41202, 41204, 41201, 41280, 41262). Comorbidities such as diabetes, hypertension, hyperlipidemia, subacromial impingement syndrome, and subacromial bursitis were also extracted and included as covariates in the multivariate analyses. Codes of these diseases were represented in **Table S7**. Demographic characteristics, including age (Field ID: 21022), sex (Field ID: 31), ethnic background (Field ID: 21000), body mass index (BMI, Field ID: 21001), Waist-to-hip ratio (W/H), multiple deprivation index (MDI, Field ID: 26410), smoking frequency (Field ID: 1249), alcohol intake frequency (Field ID: 1558), and weekly moderate and vigorous physical activity frequencies (Field ID: 884 and 904), vitamin D (Field ID: 6155) and calcium supplementation (Field ID: 6179) were extracted from 'Health-related outcomes data' and 'Online questionnaire data'. For association analyses, these demographic characteristics were regarded as potential confounding factors and included as covariates.

Age and BMI (kg/m^2) were treated as continuous variables. W/H was calculated from self-reported waist circumference (Field ID: 48) and hip circumference (Field ID: 49). Sex was coded as 0 for females and 1 for males. Ethnicity was categorized as White vs. non-White, whereas non-White referred to Asian, Black, Mixed, and other ethnic background. The MDI was included as a continuous measure of socioeconomic status. Comorbidities considered were diabetes, hypertension, hyperlipidemia, subacromial impingement syndrome, and subacromial

bursitis. All comorbidities were coded as binary variables (presence [1] vs. absence [0]), based on diagnosis records. Lifestyle factors were assessed via self-reported questionnaires. Smoking frequency was categorized as: (0) never, (1) once or twice, (2) occasionally, (3) most or all days. Alcohol intake frequency was grouped as: (0) never, (1) special occasions only, (2) 1–3 times/month, (3) 1–2 times/week, (4) 3–4 times/week, and (5) daily or almost daily. Moderate and vigorous physical activity were each categorized into: 0 days/week, 1–2 days/week, 3–5 days/week, and 6–7 days/week. Use of osteoporosis-related medication (including vitamin D, multivitamin, or none) and calcium supplementation were recorded as binary variables (yes/no).

2. Missing data handling and imputation

The missing values of the covariates were statistically analyzed. Covariate with more than 30% missing values were excluded for subsequent analysis, and the remaining missing values were interpolated using “mice” package (version 3.17.0) based on multivariate imputation by chained equation method. Imputation methods were chosen according to the variable types, such as ‘pmm’ for continuous variables (BMI, W/H, and MDI), ‘logreg’ for binary variables (calcium supplementation), ‘polyr’ for ordered factors (frequencies of smoking and alcohol intake, physical activities), and ‘polyreg’ for unordered factors (ethnic background and vitamin supplementation).

3. Sources of GWAS dataset

GWAS summary statistics of osteoporosis-related traits included a GWAS on osteoporosis risk by the Finn Biobank (8,017 cases and 391,037 controls) and multiple GWASs on BMD from

the GEFOS consortium, covering lumbar spine (N = 28,498), femoral neck (N = 32,735), forearm (N = 8,143), and skull (N = 43,800). The FinnGen Biobank GWAS on osteoporosis risk was accessed via https://www.finngen.fi/en/access_results. GEFOS datasets could be accessed via http://www.gefos.org/sites/default/files/ and https://gwas.mrcieu.ac.uk/datasets/.

The GWAS summary statistics of RCTs were derived from two independent cohort studies aimed at identifying genetic variants for RCT risk, including the UK Biobank (5,701 cases and 406,310 controls) cohort and KPNC cohort (8,357 cases and 94,622 controls). Datasets of GWAS on RCT were obtained via https://www.ebi.ac.uk/gwas/studies/GCST90044700 and https://grasp.nhlbi.nih.gov/FullResults.aspx.

All datasets were processed with standard quality control filters, including removal of SNPs with minor allele frequency < 0.01, INFO score < 0.9, or ambiguous alleles. Summary statistics were harmonized to the GRCh37/hg19 genome build, and SNPs were aligned by effect allele across datasets.

Dataset details of the data sources are provided in **Supplemental Table S8.**

4. Colocalization analysis

A colocalization analysis was conducted to identify shared genetic loci for both osteoporosis-related traits and RCTs based on the GWAS summary statistics. Prior to analysis, all SNPs were mapped to the GRCh38 genome build. SNPs present in datasets of both traits were retained for cross-trait comparison. Effect alleles were aligned and harmonized were conducted using the 'coloc' (v5.2.3) and 'TwoSampleMR' packages (v0.5.8) in R. SNPs associated with osteoporosis-related traits or RCTs (p < 5E-6) were clumped into LD blocks (pairwise r^2 >

0.001 within a 500kb window), identifying candidate loci used to assess their colocalization between osteoporosis-related traits and RCTs. SNPs within a 1000kb span centered by the lead SNPs of the candidate loci were extracted from GWAS summary statistics. A total of 234 candidate loci with osteoporosis-related traits or RCTs were screened based on a preset significance threshold of p < 5E-6, including 31 loci for osteoporosis risk, 75 for skull BMD, 40 for femoral BMD, 15 for forearm BMD, 40 for spine BMD, 15 for RCTs of the UK Biobank study, and 18 for RCTs of the KPNC study.

Effect values of the extracted SNPs were aligned by their effect alleles for the subsequent colocalization analysis. The prior probability of a SNP associated with either osteoporosis or RCTs was set at p < 1E-4, while the prior probability for a SNP associated with both diseases was set at p < 1E-5. Based on Bayesian colocalization methods, posterior probabilities were calculated for five hypotheses, including H0: No association with either disease; H1: Association with osteoporosis, not with RCTs; H2: Association with RCTs, not with osteoporosis; H3: Association with osteoporosis and RCTs but two independent SNPs; H4: Association with osteoporosis and RCTs, one shared SNP was used to determine the shared causal genetic variant. The estimated posterior probability for H4 (PP_{H4}) was specifically used to evaluate shared causality of the locus for both traits. PP_{H4} of 70% or above were chosen as strong evidence for colocalization. A PP_{H4} greater than 70% indicates a high possibility of colocalization between two traits, and a PP_{H4} between 50% to 70% indicates a moderate possibility of colocalization between two traits.

Secondary colocalization analysis with soft thresholds focusing on the identified loci was conducted to check their colocalization probabilities in other osteoporosis-related traits or RCT

risk from the other cohort. In the secondary analysis, the prior probability of a SNP associated with either osteoporosis or RCTs was set at $p < 0.05$, and the prior probability for a SNP associated with both diseases was set at $p < 0.001$.

5. Databases used in locus annotation

The colocalized loci were annotated to explore the association and functions with osteoporosis and RCTs referring to public databases, including the Ensemble (www.ensembl.org, Ensembl Release 113, October 2024), GTEx (v8, www.gtexportal.org), and Human Protein Atlas (v 24.0, www.proteinatlas.org). Regulome DB (v2.2, www.regulomedb.org) was used to analyze the regulation potential in transcription factor of the candidate locus. SNPs within ± 1 Mb of lead loci were extracted and annotated.

Supplemental results

Sensitivity analysis on the association between vitamin D supplementation and RCT risk

Due to a lack of detailed information in the questionnaires, it was unclear whether multivitamin users were actually taking preparations containing vitamin D. Specifically, vitamin supplement intake was collected via questionnaires in the UK biobank. Participants were asked "Do you regularly take any of the following?" with available options including "Vitamin D" and "Multivitamins +/- minerals". It remains unclear whether multivitamin supplements explicitly contain vitamin D.

Therefore, regular users of vitamin D supplements and users of multivitamins were either analyzed as separate groups or combined into a single exposure group. In the multivariate logistic regression model, vitamin D supplementation alone was not associated with an increased RCT risk ($p > 0.05$), whereas multivitamin use was significantly associated with increased RCT risk (adjusted OR [95% CI]: 1.09 [1.02–1.16], $p = 0.0087$). When vitamin D and multivitamin users were combined into one group representing exposure to vitamin supplementation, the association with increased RCT risk remained significant (adjusted OR [95% CI]: 1.08 [1.02–1.15], $p = 0.0118$). Although the variance inflation factor (VIF) for vitamin use was low (VIF = 1.09), indicating no serious multicollinearity, vitamin use was significantly correlated with both age and osteoporosis status ($p < 0.05$). Furthermore, inclusion of an interaction term between age and vitamin D in the multivariate model rendered the association between vitamin D use and RCT risk non-significant ($p > 0.05$), suggesting possible effect modification or confounding by age.

Supplemental Figure legends

Figure S1. Age distributions of subjects in the osteoporosis and control groups. Age distribution among osteoporotic patients (pink) and controls (blue), with each bar representing the proportion of subjects within a specific age range.

Figure S2. Receiver operating characteristic curves of multivariate Cox models. Full Cox model indicates a model with osteoporosis and covariates (red), while the nested Cox model is a model excluding osteoporosis from the full model (blue).

Figure S3. Comparison of RCT prevalence between the sexes. The height of each bar represents the percentage of RCT cases within the males (blue) and females (brown) groups in the overall cohort and age-stratified subgroups.

Figure S4. Density plots of covariates in imputed data and unimputed data. Panels A–J illustrate the distributions of selected covariates used in the multivariate analyses. Blue curves represent observed (non-missing) values, and red curves represent imputed values based on multiple imputation by chained equations method. The covariates include:(A) Waist-to-hip ratio, (B) Multiple Deprivation Index (MDI), (C) Body mass index (BMI), (D) Ethnic background, (E) Number of days per week of moderate physical activity, (F) Number of days per week of vigorous physical activity, (G) Smoking frequency, (H) Alcohol intake frequency,
(I) Vitamin supplementation intake, and (J) Calcium supplementation.

Figure S5. Love plots of standardized mean differences matched data. Each plot displays the absolute standardized mean differences of covariates between the osteoporosis and control groups before (red dots) and after (blue dots) matching. The vertical dashed line at 0.1 indicates the commonly accepted threshold for covariate balance. (A) Cross-sectional dataset using 1:5 matching; (B) Longitudinal dataset using 1:5 matching; (C) Longitudinal dataset using 1:3 matching; (D) Longitudinal dataset using 1:1 matching. Matching substantially improved covariate balance in all datasets, reducing standardized mean differences for most covariates to below 0.1.

Figure S6. Shared genetic loci for osteoporosis-related traits and rotator cuff tears (RCTs) identified through colocalization analysis. The lead SNP is marked with a purple diamond, indicating the most significant colocalization among the SNPs of the test region. The plot on the right illustrates causal variation for both traits from the same locus, while -log P values for the two traits from a single locus are presented on the left. (A, B) Co-localization analysis of GWAS data for skull BMD and

RCT; (C, D) Co-localization analysis of GWAS data for femoral neck BMD and RCT; (E, F) Co-localization analysis of GWAS data for forearm BMD and RCT.

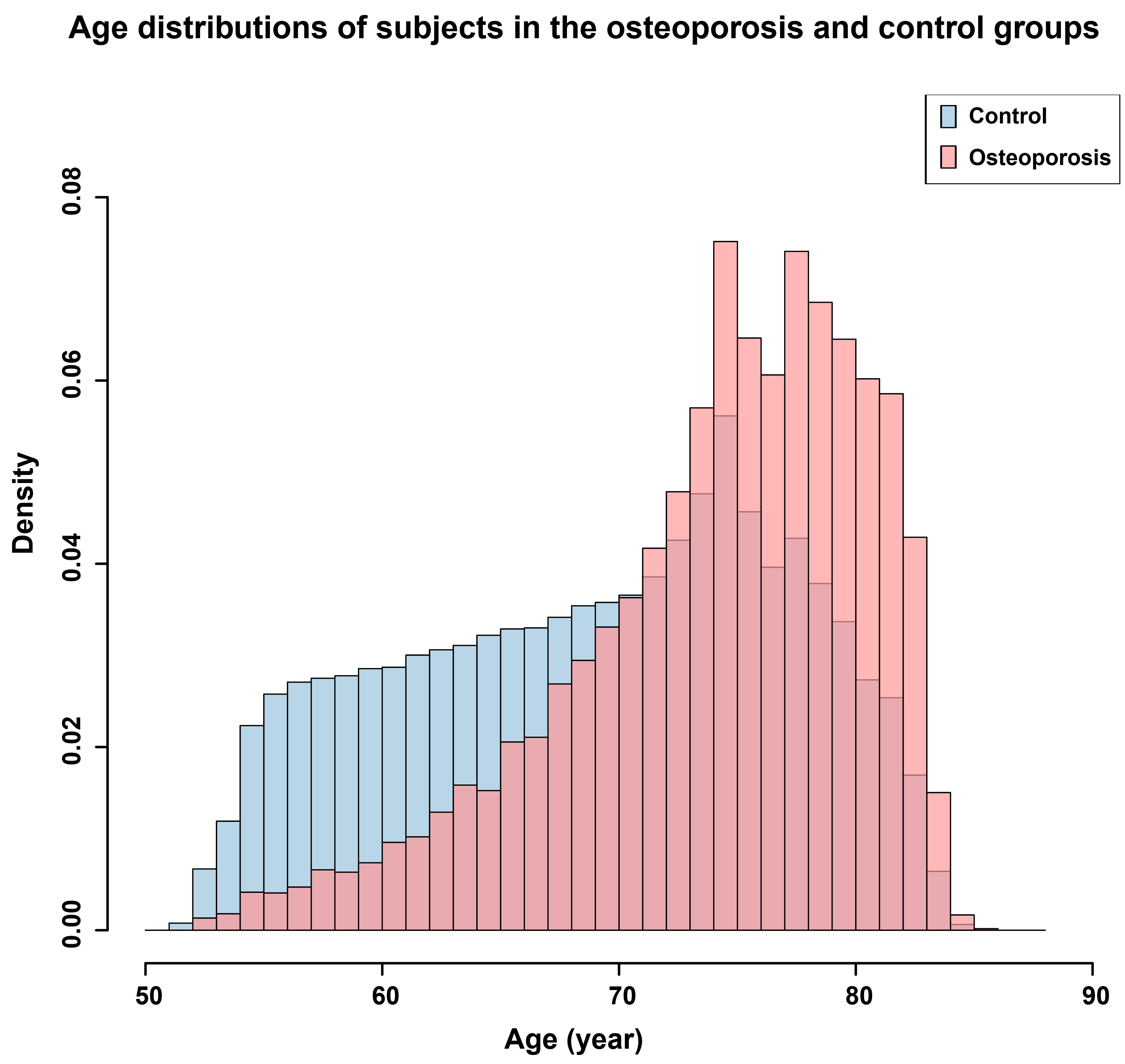

Age distributions of subjects in the osteoporosis and control groups
Control
Osteoporosis
Density
0.00
0.02
0.04
0.06
0.08
50
60
70
80
90
Age (year)

Figure S1

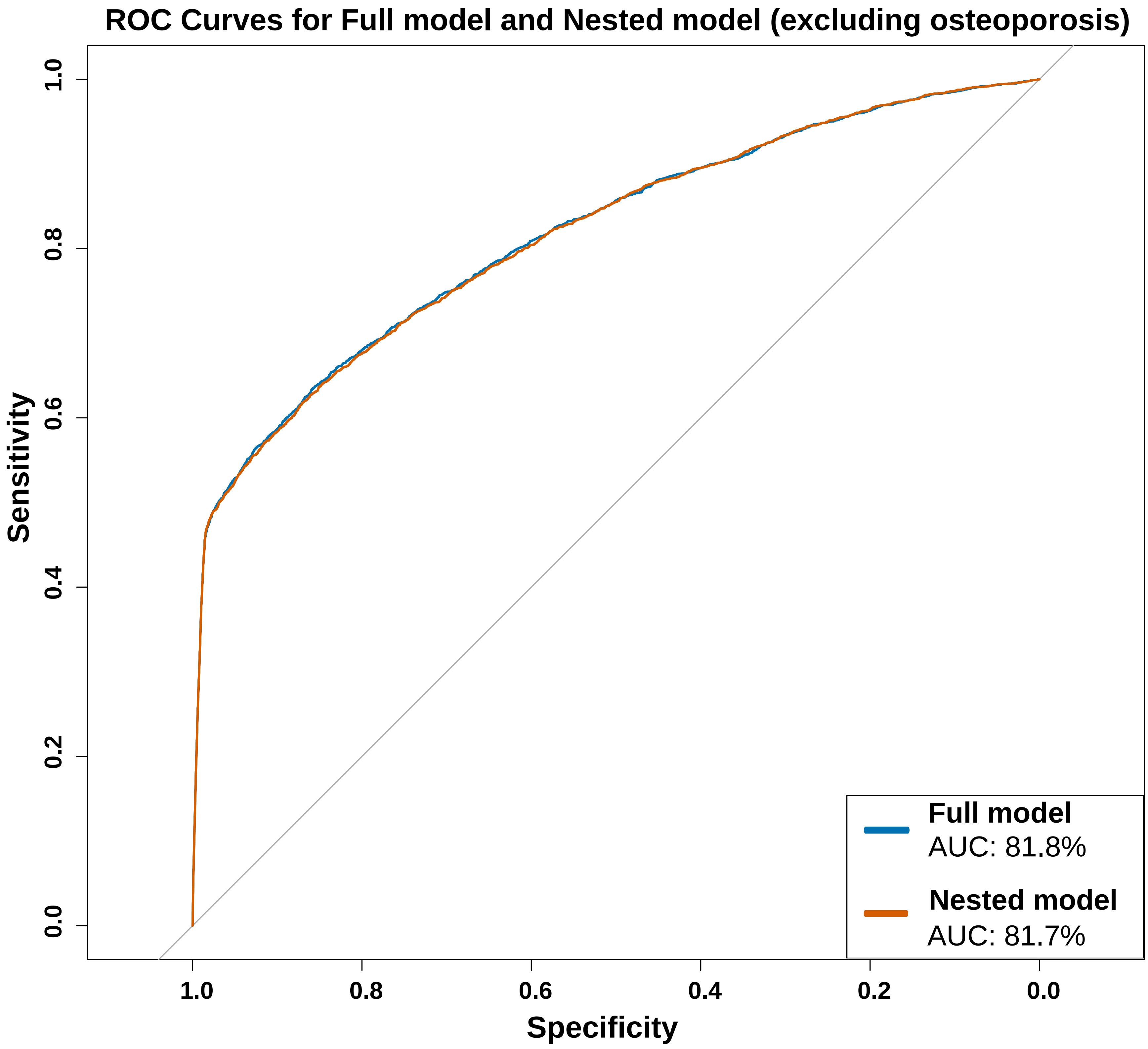
ROC Curves for Full model and Nested model (excluding osteoporosis)
Sensitivity
Specificity
1.0
0.8
0.6
0.4
0.2
0.0
Full model
AUC: 81.8%
Nested model
AUC: 81.7%

Figure S2

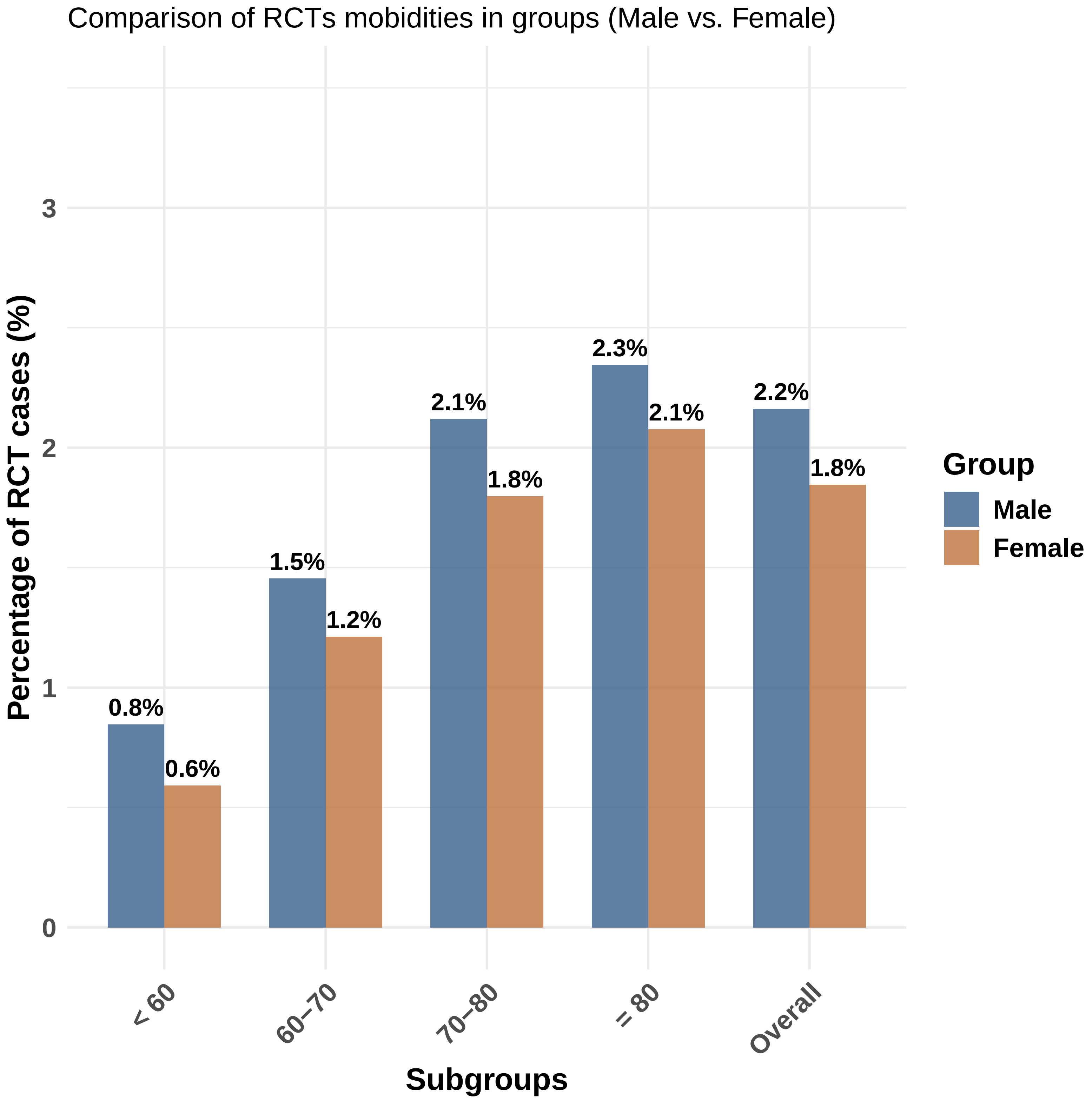
Comparison of RCTs mobidities in groups (Male vs. Female)
Percentage of RCT cases (%)
0
1
2
3
0.8%
0.6%
1.5%
1.2%
2.1%
1.8%
2.3%
2.1%
2.2%
1.8%
< 60
60–70
70–80
= 80
Overall
Subgroups
Group
Male
Female

Figure S3

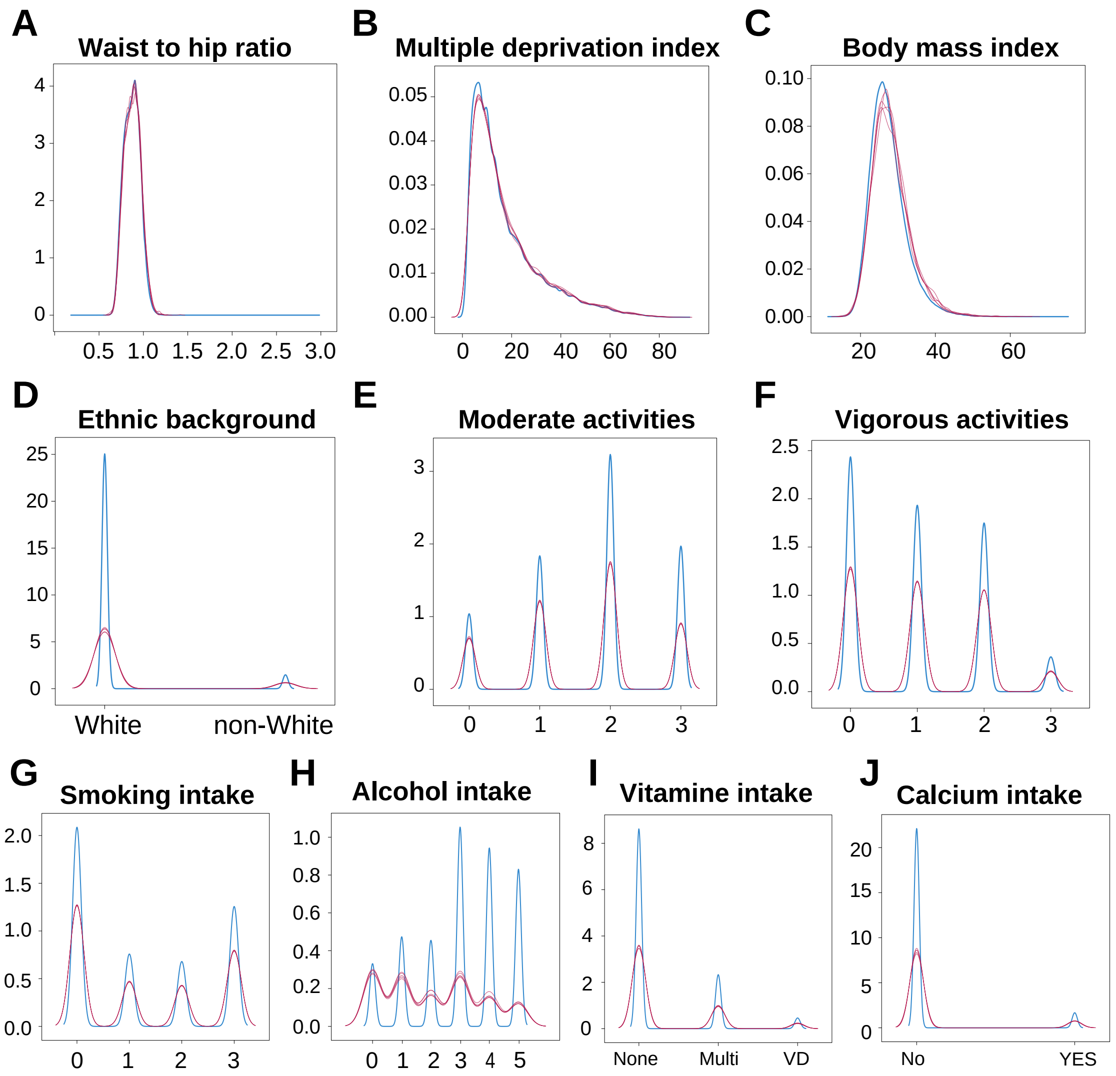
A
Waist to hip ratio
0
1
2
3
4
0.5 1.0 1.5 2.0 2.5 3.0
B
Multiple deprivation index
0.00
0.01
0.02
0.03
0.04
0.05
0 20 40 60 80
C
Body mass index
0.00
0.02
0.04
0.06
0.08
0.10
20 40 60
D
Ethnic background
0
5
10
15
20
25
White
non-White
E
Moderate activities
0
1
2
3
0 1 2 3
F
Vigorous activities
0.0
0.5
1.0
1.5
2.0
2.5
0 1 2 3
G
Smoking intake
0.0
0.5
1.0
1.5
2.0
0 1 2 3
H
Alcohol intake
0.0
0.2
0.4
0.6
0.8
1.0
0 1 2 3 4 5
I
Vitamine intake
0
2
4
6
8
None
Multi
VD
J
Calcium intake
0
5
10
15
20
No
YES

Figure S4

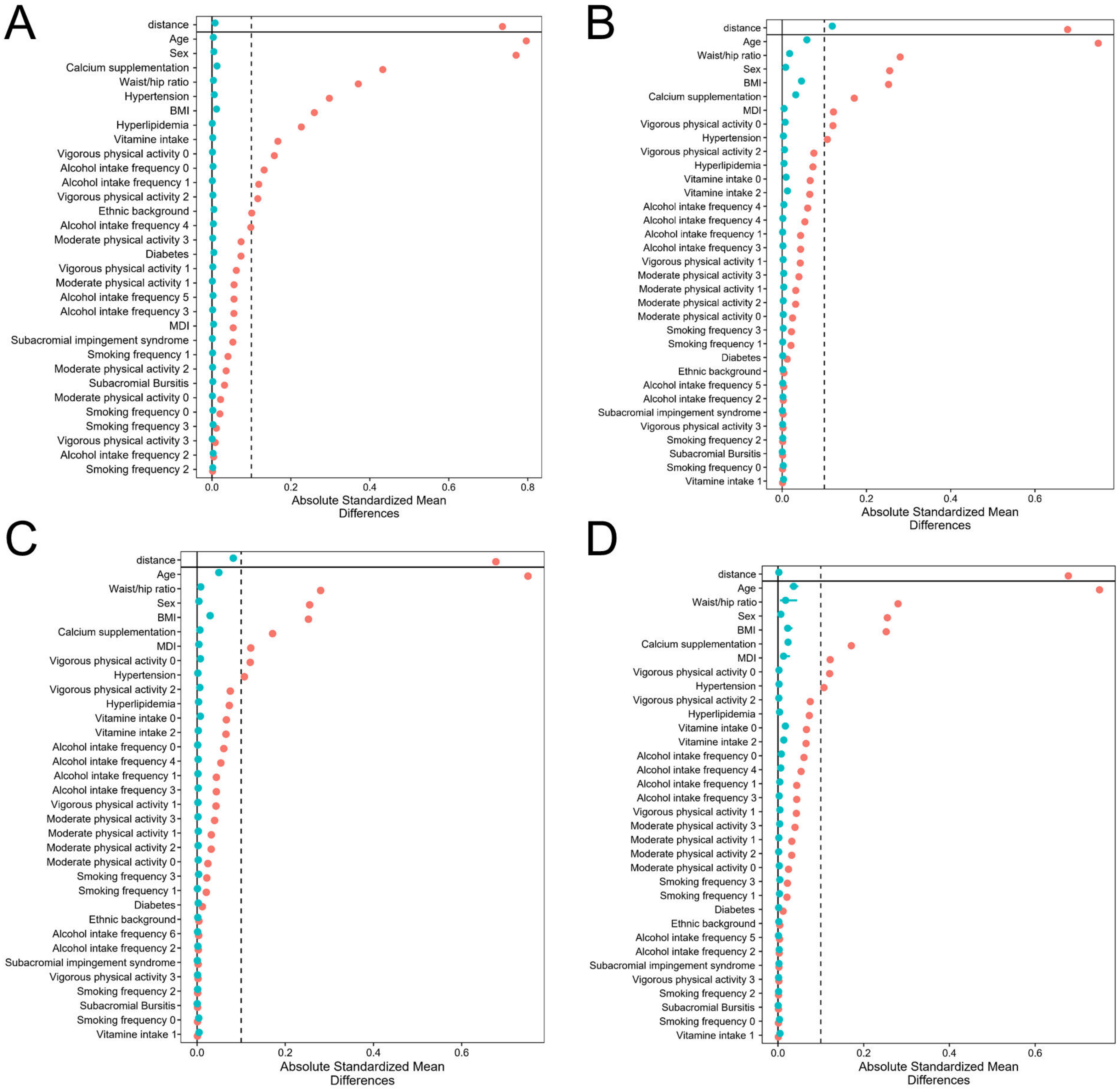
A
distance
Age
Sex
Calcium supplementation
Waist/hip ratio
Hypertension
BMI
Hyperlipidemia
Vitamine intake
Vigorous physical activity 0
Alcohol intake frequency 0
Alcohol intake frequency 1
Vigorous physical activity 2
Ethnic background
Alcohol intake frequency 4
Moderate physical activity 3
Diabetes
Vigorous physical activity 1
Moderate physical activity 1
Alcohol intake frequency 5
Alcohol intake frequency 3
MDI
Subacromial impingement syndrome
Smoking frequency 1
Moderate physical activity 2
Subacromial Bursitis
Moderate physical activity 0
Smoking frequency 0
Smoking frequency 3
Vigorous physical activity 3
Alcohol intake frequency 2
Smoking frequency 2
0.0
0.2
0.4
0.6
0.8
Absolute Standardized Mean Differences
B
distance
Age
Waist/hip ratio
Sex
BMI
Calcium supplementation
MDI
Vigorous physical activity 0
Hypertension
Vigorous physical activity 2
Hyperlipidemia
Vitamine intake 0
Vitamine intake 2
Alcohol intake frequency 4
Alcohol intake frequency 4
Alcohol intake frequency 1
Alcohol intake frequency 3
Vigorous physical activity 1
Moderate physical activity 3
Moderate physical activity 1
Moderate physical activity 2
Moderate physical activity 0
Smoking frequency 3
Smoking frequency 1
Diabetes
Ethnic background
Alcohol intake frequency 5
Alcohol intake frequency 2
Subacromial impingement syndrome
Vigorous physical activity 3
Smoking frequency 2
Subacromial Bursitis
Smoking frequency 0
Vitamine intake 1
0.0
0.2
0.4
0.6
Absolute Standardized Mean Differences
C
distance
Age
Waist/hip ratio
Sex
BMI
Calcium supplementation
MDI
Vigorous physical activity 0
Hypertension
Vigorous physical activity 2
Hyperlipidemia
Vitamine intake 0
Vitamine intake 2
Alcohol intake frequency 0
Alcohol intake frequency 4
Alcohol intake frequency 1
Alcohol intake frequency 3
Vigorous physical activity 1
Moderate physical activity 3
Moderate physical activity 1
Moderate physical activity 2
Moderate physical activity 0
Smoking frequency 3
Smoking frequency 1
Diabetes
Ethnic background
Alcohol intake frequency 6
Alcohol intake frequency 2
Subacromial impingement syndrome
Vigorous physical activity 3
Smoking frequency 2
Subacromial Bursitis
Smoking frequency 0
Vitamine intake 1
0.0
0.2
0.4
0.6
Absolute Standardized Mean Differences
D
distance
Age
Waist/hip ratio
Sex
BMI
Calcium supplementation
MDI
Vigorous physical activity 0
Hypertension
Vigorous physical activity 2
Hyperlipidemia
Vitamine intake 0
Vitamine intake 2
Alcohol intake frequency 0
Alcohol intake frequency 4
Alcohol intake frequency 1
Alcohol intake frequency 3
Vigorous physical activity 1
Moderate physical activity 3
Moderate physical activity 1
Moderate physical activity 2
Moderate physical activity 0
Smoking frequency 3
Smoking frequency 1
Diabetes
Ethnic background
Alcohol intake frequency 5
Alcohol intake frequency 2
Subacromial impingement syndrome
Vigorous physical activity 3
Smoking frequency 2
Subacromial Bursitis
Smoking frequency 0
Vitamine intake 1
0.0
0.2
0.4
0.6
Absolute Standardized Mean Differences

Figure S5

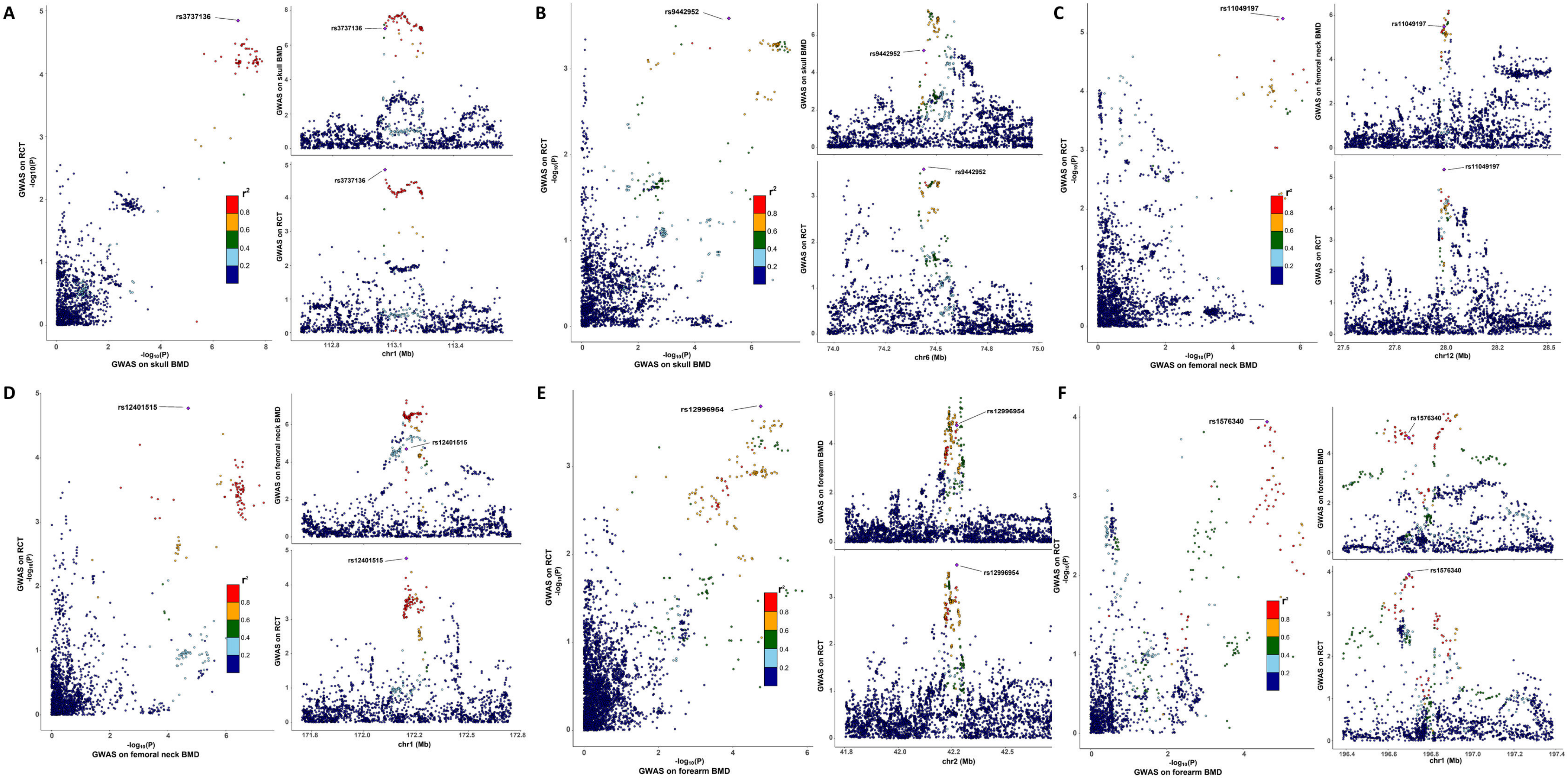

A
rs3737136
GWAS on RCT -log10(P)
-log10(P)
GWAS on skull BMD
GWAS on skull BMD
GWAS on RCT
chr1 (Mb)
r2
B
rs9442952
GWAS on skull BMD
chr6 (Mb)
C
rs11049197
GWAS on femoral neck BMD
chr12 (Mb)
D
rs12401515
GWAS on femoral neck BMD
chr1 (Mb)
E
rs12996954
GWAS on forearm BMD
chr2 (Mb)
F
rs1576340
GWAS on forearm BMD
chr1 (Mb)

Figure S6

Table S1. Comparison of RCT incidence and percentage in groups (Osteoporosis vs. Control)

Categories	Osteoporosis		Control		Chi-square	P-value
	RCT incidence	(%)	RCT incidence	(%)		
**Age < 60**	15	1.77	540	0.70	12.19	0.0005
**60 ≤ Age < 70**	107	2.35	1827	1.30	36.41	1.59E-09
**70 ≤ Age < 80**	358	2.60	3593	1.96	25.68	4.03E-07
**≥ 80**	124	2.97	740	2.22	9.04	0.0026
**Female**	494	2.54	3201	1.36	175.84	3.93E-40
**Male**	110	2.79	3499	1.76	22.76	1.83E-06
**Overall**	604	2.59	6700	1.54	153.10	3.64E-35

Table S2. Logistic regression analysis on osteoporosis and RCTs in unimputed dataset

Model	Variables	OR (95%CI)	P-value
**Univariate model**	**Osteoporosis**	1.69 (1.56-1.84)	1.49E-34
**Multivariate model**	**Osteoporosis**	1.38 (1.23-1.54)	5.58E-08
	**Age**	1.04 (1.04-1.05)	7.82E-74
	**Sex (Female)**	0.88 (0.80-0.95)	0.0019
	**Ethnic backgroud (non-White)**	1.44 (1.26-1.64)	5.67E-08
	**W/H ratio**	1.76 (1.05-2.93)	3.11E-02
	**BMI**	1.03 (1.03-1.04)	9.69E-21
	**Hypertension**	1.34 (1.25-1.43)	1.11E-17
	**Hyperlipidemia**	1.24 (1.15-1.33)	5.23E-09
	**Impingement syndrome**	50.15 (47.07-53.43)	< 1E-300
	**Bursitis**	5.26 (4.60-6.02)	7.93E-129
	**Smoking**	1.06 (1.01-1.12)	0.0323
	**Moderate physical activity**	1.17 (1.08-1.27)	0.0001
	**Vigorous physical activity**	1.20 (1.10-1.32)	8.18E-05
	**Vitamin supplementation**		
	Multivitamin	1.11 (1.04-1.20)	0.0030
	Vitamin D	1.07 (0.92-1.24)	0.3853

OR, odds ratio. CI, confidence interval. W/H ratio, waist to hip ratio. BMI, body mass index.

Table S3. Demographic characteristics of study subjects in longitudinal analysis

Group	Osteoporosis<br>Mean ± SD<br>Count (%)	Osteopenia<br>Mean ± SD<br>Count (%)	Controls<br>Mean ± SD<br>Count (%)
**Samplesize**	6,588	70,862	187,569
**Age**	74.73 ± 6.60	71.51 ± 7.73	69.78 ± 8.11
**Sex**			
Femal	4,891 (74.2%)	45,715 (64.5%)	91,382 (48.7%)
Male	1697 (25.8%)	25,147 (35.5%)	96,187 (51.3%)
**BMI**	26.30 ± 5.44	26.87 ± 4.54	27.70 ± 4.77
**W/H ratio**	0.85 ± 0.09	0.86 ± 0.09	0.88 ± 0.09
**Ethnicity (White)**	6,324 (96.0%)	69,017 (97.4%)	179,287 (95.6%)
**Related comorbidities**			
Diabetes	776 (11.8%)	6,365 (9.0%)	19,835 (10.6%)
Hypertension	2,869 (43.5%)	23,359 (33.0%)	61,547 (32.8%)
Hyperlipidemia	1,604 (24.3%)	12,099 (17.1%)	31,946 (17.0%)
ubacromial impingement syndrome	142 (2.2%)	1,191 (1.7%)	3,591 (1.9%)
Subacromial Bursitis	45 (0.7%)	356 (0.5%)	1,047 (0.6%)
**Smoking frequency**			
Most or all days	1,655 (25.1%)	17,590 (24.8%)	44,777 (23.9%)
Occasionally	810 (12.3%)	9,318 (13.1%)	24,339 (13.0%)
Once or twice	815 (12.4%)	9,766 (13.8%)	28,159 (15.0%)
Never	2,551 (38.7%)	27,265 (38.5%)	76,453 (40.8%)
**Alcohol intake frequency**			
Daily or almost daily	1,312 (19.9%)	14,959 (21.1%)	38,093 (20.3%)
3-4 times/week	1,251 (19.0%)	15,902 (22.4%)	45,807 (24.4%)
1-2 times/week	1,477 (22.4%)	17,965 (25.4%)	50,303 (26.8%)
1-3 times/month	703 (10.7%)	7,964 (11.2%)	20,605 (11.0%)
Special occasions	974 (14.8%)	8,413 (11.9%)	19,535 (10.4%)
Never	855 (13.0%)	5,531 (7.8%)	12,963 (6.9%)
**Moderate physical activity**			
0 day/week	958 (14.5%)	9,311 (13.1%)	23,118 (12.3%)

1-2 day/week	1,220 (18.5%)	15,410 (21.7%)	42,249 (22.5%)
3-5 day/week	2,236 (33.9%)	26,203 (37.0%)	71,803 (38.3%)
6-7 day/week	1,674 (25.4%)	15,884 (22.4%)	41,458 (22.1%)
**Vigorous physical activity**			
0 day/week	2,943 (44.7%)	27,926 (39.4%)	63,416 (33.8%)
1-2 day/week	1,547 (23.5%)	19,679 (27.8%)	54,124 (28.9%)
3-5 day/week	1,238 (18.8%)	15,882 (22.4%)	50,969 (27.2%)
6-7 day/week	327 (5.0%)	3,302 (4.7%)	10,040 (5.4%)
**Multiple deprivation Index**	18.33 ± 14.91	16.69 ± 14.02	16.48 ± 13.88
**Vitamine D supplementation**			
Vitamin D	628 (9.5%)	2,978 (4.2%)	5,735 (3.1%)
Multivitamin	1,326 (20.1%)	14,397 (20.3%)	37,690 (20.1%)
**Calcium supplementation**	1,458 (22.1%)	5,506 (7.8%)	9,434 (5.0%)

Table S4. Cox regression analysis on osteoporosis and RCTs in unimputed dataset

Model	Variable	HR (95%CI)	P-value
Univariate model	**Osteoporotic condition**		
	Osteopenia	1.00 (0.92-1.08)	0.9160
	Osteoporosis	1.79 (1.49-2.14)	2.33E-10
Multivariate model	**Osteoporotic condition**		
	Osteopenia	1.06 (0.97-1.16)	0.2209
	Osteoporosis	1.60 (1.31-1.94)	2.74E-06
	**Age**	1.03 (1.02-1.03)	4.62E-24
	**Ethnic backgroud (non-White)**	1.44 (1.18-1.75)	0.0003
	**BMI**	1.02 (1.02-1.03)	4.30E-08
	**W/H ratio**	2.09 (1.28-3.39)	0.0030
	**Hypertension**	1.37 (1.25-1.49)	1.33E-12
	**Hyperlipidemia**	1.22 (1.11-1.34)	1.95E-05
	**Impingement syndrome**	38.65 (35.53-42.04)	< 1E-300
	**Bursitis**	2.85 (2.52-3.23)	8.05E-64
	**Moderate physical activity**	1.17 (1.06-1.30)	0.0043
	**Vigorous physical activity**	1.12 (0.99-1.26)	0.0709
	**Vitamin supplementation**		
	Multivitamin	1.12 (1.02-1.23)	0.0163
	Vitamin D	0.97 (0.79-1.19)	0.7669

HR, hazard ratio. CI, confidence interval. W/H ratio, waist to hip ratio. BMI, body mass index.

Table S5. LDSC analysis on genetic correlation between osteoporosis and RCTs.

Traits	Heritability		Genetic correlation estimation			
			RCTs in the UK biobank		RCTs in the KPNC cohort	
	H2 ± SE	P value	rG ± SE	P value	rG ± SE	P value
**Osteoporosis**	0.008 ± 0.002	1.43E-07	-0.04 ± 0.11	0.7354	0.16 ± 0.15	0.2628
**Skull BMD**	0.253 ± 0.023	2.97E-27	-0.02 ± 0.02	0.3223	0.02 ± 0.03	0.5858
**Femoral neck BMD**	0.097 ± 0.013	2.23E-13	0.18 ± 0.10	0.0703	0.14 ± 0.11	0.1994
**Spine BMD**	0.098 ± 0.014	1.05E-12	0.01 ± 0.09	0.9061	-0.12 ± 0.13	0.3572
**Forearm BMD**	0.072 ± 0.039	0.06797257	0.03 ± 0.21	0.8940	0.35 ± 0.28	0.2074
**RCTs in the UK biobank**	0.008 ± 0.001	5.71E-12	-			
**RCTs in the KPNC cohort**	0.014 ± 0.004	5.78E-04	-			

Table S6. Annotation of the shared loci of osteoporosis and RCTs

Locus	Position	Nearest gene	Consequence	Reported associated phenotypes	Reference (PMID)
rs3737136	chr1:112517810	WNT2B	intron variant	Cortical area measurement [1], body height [2], blood pressure [3]	[1]33875891; [2]37106081; [3]35762941
rs9442952	chr6:73728266	CD109	intron variant	Heel bone mineral density [4]	[4]30595370
rs11049197	chr12:27843799	KLHL42	-	Dysplasia of the hip[5]	[5]36902448
rs12996954	chr2:42046107	PKDCC	2kb upstream variant	Bone mineral density[6]	[6]37500982
rs1576340	chr1:196729581	CFH	genic downstream transcript variant	Age-related macular degeneration[7	[7]21882633
rs12401515	chr1:172234360	DNM3	genic downstream transcript variant	-	-

Table S7. Coding of diseases in health care systems

Disease	ICD-10	Read2	Read3
**Osteoporosis**	M80, M81, M82	N3312, N3313, N3314, N3315, N3316, N3318, N3319, N331A, N331B, N331H, N331J, N331K, N331L, N331M, NyuB0, NyuB8, N330., N3300, N3301, N3302, N3303, N3304, N3305, N3306, N3307, N3308, N330B, N330C, N330D, N330z, N3370, NyuB1, N330A, NyuB2	N3303, N3305, N3371, N3301, N3304, N330., N3302, N3306, N3307, N3308, N330A, N330B, N3312, N3313, N3314, N3315, N3316, N3318, N3319, N331A, N331B, NyuB0, N3309
**Rotator cuff tear**	M75.1	N2115, S5Q0, S5Q2, S5Q1, 7H530, 7H534, 7H535, 7H536, 7H537, 7H538	X600L, Xa9Cd, Xa9Wn, Xa9Wo, XaBDZ, XaPSk, XaPSL, XaPSM, XaPSN, XaYi8, 7H530, S5Q0, S5Q2, S5Q1, N2115
**Subacromial impingement syndrome**	M75.4	N2122, N2124	N2122, N2124
**Subacromial bursitis**	M75.5	N2116, N2118	N2116, N2118
**Diabetes**	E10, E11, E12, E13, E14	C1000, C1001, C100z, C101., C1010, C1011, C101y, C101z, C102., C1020, C1021, C102z, C103., C1030, C1031, C103y, C103z, C104y, C105., C1050, C1051, C105y, C105z, C106., C1060, C1061, C106y, C106z, C107., C1070, C1071, C1072, C1073, C1074, C107y, C107z, C108., C1080, C1081, C1082, C1083, C1084, C1085, C1086, C1088, C1089, C108A, C108E, C108y, C108z, C109., C1090, C1091, C1092, C1093, C1094, C1095, C1097, C1099, C109D, C109J, C109K, C10A., C10A0, C10A1, C10A2, C10A3, C10A4, C10A5, C10A6, C10A7, C10AW, C10AX, C10B., C10B0, C10C., C10D., C10E., C10E0, C10E1, C10E2, C10E3, C10E4, C10E5, C10E6, C10E8, C10E9, C10EA, C10EE, C10EM, C10EN, C10ER, C10F., C10F0, C10F1, C10F2, C10F3, C10F4, C10F5, C10F7, C10F8, C10F9, C10FD, C10FJ, C10FK, C10FN, C10FP, C10FS, C10G., C10G0, C10H., C10H0, C10J., C10J0, C10K., C10K0, C10L., C10L0, C10M., C10M0, C10N., C10N0, C10N1, C10P0, C10P1, C10Q., C10y., C10y0, C10y1, C10yy, C10yz, C10z., C10z0, C10z1, C10zy, C10zz, C11y0,	C1000, C1001, C100z, C101y, C101z, C102z, C103y, C103z, C105z, C106., C1060, C106z, C107., C107z, C108y, C108z, C10yz, C10zz, X008t, X40JI, X40KH, XaJlL, XaJlN, XaJlO, XaJlR, XaMzI, Xaagd, XacoB
**Hypertension**	I10	G2..., G20.., G200., G201., G202., G203., G20z., G25.., G250., G251., G26.., G27.., G28.., G2y.., G2z..	G20z., G2y.., G2z.., XSDSb, XaIWn, XaZzo

**Hyperlipidemia**	E78	C32.., C320., C3200, C3201, C3202, C3203, C3204, C3205, C3206, C320y, C320z, C321., C3210, C322., C3220, C323., C324., C325., C3250, C3251, C3252, C3253, C325z, C328., C329., C32y., C32yz, C32z., Cyu8D, Cyu8E	C32.., C320., C3201, C3202, C320y, C320z, C321., C322., C323., C324., C325., C3250, C3251, C3252, C3253, C325z, C32y., C32yz, C32z., Cyu8D, Cyu8E, X003s, X00dK, X20DV, X20DW, X40VH, X40VI, X40VT, X40VW, X40VY, X40Vd, X40Vf, X40Vg, X40Vh, X40Vi, X40Vm, X40Vn, X40Vo, X40Vp, X40Vq, X40Vs, X40Vt, X40Wx, X40Wy, X40Wz, X40X0, X40X1, X40X2, X40X3, X40X5, X40X6, X40X7, X40X9, X40XA, X40XD, X40XH, X40XI, X40XO, X40XP, X40XQ, X40XR, X40XU, X40XV, X40XW, X40XX, X40XY, X40XZ, X40Xa, X40Xb, X40Xc, X40Xd, X40Xe, X40Xf, X40YU, X50DQ, X50Fh, X50Fi, X50Fm, X50Fp, X76tz, X78Tl, X78qa, XE11R, XE11T, XE11U, XE11V, XE11W, Xa9At, XaL5p, XaR4h, XaR4i, XaR4k, XaRG5

Table S8. Data sources of GWAS summary statistics

Trait	Consortium	Source database	Release year	Major population	Sample size	nSNP	Reference(PMID)
Osteoporosis	Finn Biobank	https://r10.finngen.fi/	2023	Caucasian	399,054	21,306,139	-
Skull BMD	GEFOS	http://www.gefos.org/sites/default/files/	2023	Caucasian	43,800	10,040,455	37402774
Lumbar spine BMD	GEFOS	https://gwas.mrcieu.ac.uk/datasets/ieu-a-982/	2015	Caucasian	44,731	9,282,330	-
Femoral neck BMD	GEFOS	https://gwas.mrcieu.ac.uk/datasets/ieu-a-980/	2015	Caucasian	49,988	9,280,933	-
Forearm BMD	GEFOS	https://gwas.mrcieu.ac.uk/datasets/ieu-a-977/	2015	Caucasian	10,805	9,095,196	-
Rotator cuff tear	UK Biobank	https://www.ebi.ac.uk/gwas/studies/GCST90044700	2021	Caucasian	412,011	7,836,690	32663566
Rotator cuff tear	KPNC	https://grasp.nhlbi.nih.gov/FullResults.aspx	2017	Caucasian	102,979	7,411,676	29228018
